# Higher Affinity Antibodies Bind With Lower Hydration and Flexibility in Large Scale Simulations

**DOI:** 10.3389/fimmu.2022.884110

**Published:** 2022-05-30

**Authors:** Mabel T. Y. Wong, Sebastian Kelm, Xiaofeng Liu, Richard D. Taylor, Terry Baker, Jonathan W. Essex

**Affiliations:** ^1^ School of Chemistry, University of Southampton, Southampton, United Kingdom; ^2^ UCB, Slough, United Kingdom

**Keywords:** antibody-antigen interactions, antibody binding, antibody affinity, antibody interface hydration, CDR flexibility, molecular dynamics, replica exchange

## Abstract

We have carried out a long-timescale simulation study on crystal structures of nine antibody-antigen pairs, in antigen-bound and antibody-only forms, using molecular dynamics with enhanced sampling and an explicit water model to explore interface conformation and hydration. By combining atomic level simulation and replica exchange to enable full protein flexibility, we find significant numbers of bridging water molecules at the antibody-antigen interface. Additionally, a higher proportion of interactions excluding bulk waters and a lower degree of antigen bound CDR conformational sampling are correlated with higher antibody affinity. The CDR sampling supports enthalpically driven antibody binding, as opposed to entropically driven, in that the difference between antigen bound and unbound conformations do not correlate with affinity. We thus propose that interactions with waters and CDR sampling are aspects of the interface that may moderate antibody-antigen binding, and that explicit hydration and CDR flexibility should be considered to improve antibody affinity prediction and computational design workflows.

## 1 Introduction

Antibodies are increasingly attractive biological drugs, and at present are at the forefront of vaccine and therapeutic developments against COVID-19, a pandemic that has caused over 5.5 million deaths as of January 2022 (1, 2). The majority of an antibody’s high affinity and specificity is dictated *via* six hypervariable loops at the antigen binding site, known as Complementarity Determining Regions (CDRs) (3, 4). This makes antibodies ideal for treating diseases, but their binding is difficult to predict and understand at the atomic level.

Therapeutic antibodies are currently generated by animal immunization followed by hybridoma or B-cell selection technology, or by display methods (5–7). Structure-based rational design however offers additional insight and benefits including epitope specificity, mechanisms of action, and affinity maturation. A proof-of-concept example demonstrating the advantage of atomistic structure-based design was carried out by the Vendruscolo group, who designed complementary peptides against different parts of an amyloid-β peptide (8, 9). Grafting these designs onto single domain heavy chain CDR3s, the rate of amyloid-β peptide aggregation during *in vivo* validation was only affected when specific parts of the amyloid peptide were targeted. Additionally, rational design of a therapeutic antibody was recently exemplified in the case of an anti-IL-17, Bimekizumab, where a computational affinity maturation method was used to provide neutralization potency against both IL-17A and IL-17F isoforms (10).

Computational methods offer a solution to modern structure-based rational antibody design. There have been several *in silico* attempts at *de novo* design: OptMAVEn (11), AbDesign (12), hotspot grafting with CDR-H3 swapping (13), and re-epitoping (14). OptMAVEn and AbDesign use a jigsaw-like approach, assembling fragments of antibody variable region structures. The most notable differences lie in how the variable regions are fragmented, the in-house protocols to optimize and refine designs, and the scoring functions used to predict binders. AbDesign shares the same scoring function as Liu et al.’s attempt of hotspot grafting and CDR-H3 swapping, applied to proof-of-concept target Keap1 (13). This target has a solved structure with native binder Nrf2. *In silico* alanine scanning identified Nrf2 residues that contribute strongly to the Keap1-Nrf2 interaction, and antibody scaffolds were selected based on their ability to replicate the hotspots’ conformation, allowing Nrf2’s binding motif to be grafted onto a CDR. This was followed by a round of swapping out the selected antibody’s H3 with other known H3 structures.

Re-epitoping is the only structure-based rational design protocol without a stage of piecing together different antibody fragments (14). Instead, potential binders are docked against antigens and ranked by the agreement between docking programs’ models and predicted interfacial contacts. The best model’s mutations to CDRs were assessed by scoring and molecular dynamics to determine their effect on the model’s stability. Libraries were designed for mutations at specific positions determined by the scoring, which were then screened by experimental display methods. Although the final stage here is purely experimental, the previously mentioned protocols also required some form of manual interference, sometimes even between *in silico* stages, to filter out false positives (15, 16). In all cases, false negatives may have also been missed, making the success rates for these protocols even more difficult to compare when they are reported.

This clear dependence on experimental validation suggests that current scoring functions do not account for all affinity-determining aspects of antibody-antigen interactions. One feature these methods share is the use of implicit solvent models when scoring their designs – OptMAVEn, AbDesign, and Liu et al. use the Lazaridis-Karplus model, whereas re-epitoping utilizes the Generalized Born using Molecular Volume model (17, 18). Without the use of explicit waters, any indirect interactions formed *via* individual bridging waters will be poorly described using an implicit model. A second common feature is the use of static crystal structures or models, despite the potential for antibodies to undergo conformational change upon antigen binding (19).

Previous work to simulate these interfaces and study conformations did not focus on antibody design. Scoring of antibody-antigen molecular dynamics simulations by MM-PBSA/GBSA methods either struggled to distinguish poorly docked poses from crystal structure ones, or only used neutralizing activity data as opposed to kinetic data when comparing predicted binding energies (20, 21). Methods involving enhanced sampling come closer to scoring different designs, such as combining free energy perturbation with Replica Exchange with Solute Tempering for an *in silico* alanine scan on antibodies (22). The authors achieved an R^2^ of 0.49 for their predicted binding energies with experiment, although all fifty-five mutations tested did not change the side chain’s charge. The Liedl group ran metadynamics on antigen bound CDRs and produced Markov state models for their conformational populations. They proposed a correlation between specificity and lower H3 flexibility, and that CDRs interchange between canonical structures on the µs to ms timescale (23, 24). We wish to further explore how CDRs behave without their antigens and how they behave when all subjected to increased sampling, as well as relate their behavior to antibody affinity.

To further our understanding on the potential role of water and CDR flexibility at these interfaces, we have carried out a sophisticated simulation study at the highest level of theory feasible to maximize our resources. Replica Exchange with Solute Scaling (REST2), a cutting edge advanced molecular dynamics technique that is rarely applied to antibodies, was used to enable large scale sampling of the CDRs and capture waters explicitly in simulation (25). REST2 simulations do not require prior knowledge of CDR behavior, allowing their full flexibility to be explored by weakening interactions these loops experience with their environment. We analyzed antibody-antigen interfaces in a varied dataset of carefully curated crystal structures, selected to be representative of those deposited in the Protein Data Bank. CDR sampling was also quantified, and different aspects of this analysis were compared to the antibodies’ experimental affinities. The analyses illustrate that these interfaces involve many waters and provide evidence of a potential link between water, CDR flexibility, and an antibody’s affinity. We thus propose that these aspects play an important role in antibody binding and need explicit consideration in antibody design.

## 2 Materials and Methods

### 2.1 Starting Structures

Nine antibodies’ structures were chosen using the SAbDab database’s non-redundant search, ensuring a range of sequences were found in the selection (26). Structures were initially filtered by the following criteria: the antigen was a protein but not a short peptide; the antibody was solved in complex with and without its antigen; structures had no missing residues in the variable (Fv) region; and CDR sequences were identical in both structures. From this initial selection, therapeutic antibodies and structures under 2.5 Å resolution were prioritized, and binding affinities were considered to ensure a wide range in the dataset. The details of the filtering process are described fully in the **Supplementary Methods**. The final structures are listed in **Table 1**.

**Table 1:** Antibody-antigen dataset structures. Names for referring to antibodies in the results and discussion are given in parentheses.

Antibody	Antibody-Antigen PDB	Antibody only PDB	K_d_ (nM)
5J8 (Anti-H1N1)	4m5z (27)	4m5y (27)	26 (28)
125-2H (Anti-IL-18)	2vxt (29)	2vxu (29)	0.533 (30)
AL-57 (Anti-LFA)	3hi6 (31)	3hi5 (31)	4700 (32)
3M4E5 (Anti-MHC)	3gjf (33)	3gje (33)	46 (33)
9F8 (Anti-ObR)	3v6o (34)	3vg0 (34)	Not available
Canakinumab (Anti-IL-1βa)	4g6j (35)	4g5z (35)	0.0305 (36)
Certolizumab (Anti-TNFα)	5wux (37)	5wuv (37)	0.09 (38)
Gevokizumab (Anti-IL-1βb)	4g6m (35)	4g6k (35)	0.0003 (39)
Pembrolizumab (Anti-PD)	5ggs (40)	5dk3 (41)	0.029 (42)

Only the antibody’s Fv region, the antigen where applicable, and crystal waters were kept for simulation. Missing residues were modelled using MODELLER version 9.19 (43). The N- and C-termini were capped with acetyl and amide groups respectively, and structures had protonation states assigned for pH 7.4 using HTMD 1.12.2 (44). Histidine protonation states were determined on a case-by-case basis, with the aim of maximizing hydrogen bonding.

### 2.2 Simulation Details

Although molecular dynamics can model the time evolution of a system, extensive exploration of CDR conformational populations would not be achievable within reasonable timescales. We thus turn to enhanced sampling methods such as Replica Exchange with Solute Scaling (REST2), whereby interactions between atoms are weakened by a ratio (λ) during molecular dynamics, increasing the rate they sample different conformations (25). An acceptance test ensures that only conformations compatible with fully interacting atoms are included in the final trajectory that is analyzed.

All simulations were performed using GROMACS 2018.2 patched with PLUMED 2.4.2 (45–48). Structures were modelled by the Amber ff14SB force field except for the Mn^2+^ ion in PDB 3hi6, which was modelled using parameters by Bradbook et al. (49, 50). Distance restraints of 1000 kJ mol^-1^ nm^2^ were applied between the Mn^2+^ ion and coordinating antigen atoms. Cubic boxes with edges 1.2 and 1.8 nm away from the protein were used for Fv only (apo) and Fv-antigen (holo) simulations respectively, with periodic boundary conditions applied in all three directions. Boxes were solvated with TIP3P water and 0.15 M NaCl, including ions to neutralize the system (51). This generated systems of 55,000-73,000 atoms for the apo setup, and 149,000-326,000 atoms for the holo setup depending on the size of the antigen. The energy of each system was minimized using steepest descent and conjugate gradient for 5000 steps each.

Prior to generating the scaled replicas, systems were equilibrated for 50 ps under NVT conditions and 200 ps under NPT conditions. Temperature was maintained at 300 K using the velocity-rescaling thermostat with a time constant of 0.1 ps, and pressure at 1 bar using the Berendsen barostat and a time constant of 2 ps (52, 53). The leapfrog integrator with a 2 fs timestep was used (54). Both non-bonded van der Waals and electrostatic Coulomb interactions had a 0.8 nm cut-off, using the Verlet cut-off scheme and long range dispersion corrections for energy and pressure. The particle mesh Ewald algorithm was used for long range electrostatics with a Fourier spacing of 0.1 nm and cubic interpolation to assign charges to the grid (55). Hydrogen-containing bonds were constrained using LINCS (56).

To prevent proteins from unfolding due to REST2’s weakened interactions, we specifically scale down CDR residues. Replicas with geometrically distributed λ values between 0.35 and 1 were generated from the equilibrated structures, with the minimum λ determined by preliminary work to ensure sufficient CDR sampling was taking place. Twenty-four replicas were used for holo systems and twenty for apo, chosen to keep acceptance probabilities between 20-40% for as much of the dataset as possible. CDR residues, selected by their Chothia numbering as in the AbDb database, had their inter- and intra-molecular interactions scaled (57, 58). All six CDRs on an antibody were scaled simultaneously, as preliminary work suggested that their conformations were dependent on each other. Each replica underwent a second NVT equilibration for 1 ns under the same conditions as the first NVT, but with a Nosé-Hoover thermostat and time constant of 2 ps, as well as increased van der Waals and electrostatic cut-offs to 1.0 nm and no dispersion correction (59, 60). This was followed by 100 ns of REST2 under the same simulation conditions, with frames deposited every 10 ps in output trajectories. This culminated in 40 µs of simulations for the entire dataset.

### 2.3 Analysis

In REST2, the interface behavior of interest is found in the unscaled replica, which has an ensemble equivalent to that of unbiased MD. Each trajectory was aligned by backbone atoms making up the Fv’s heavy and light chain (V_H_/V_L_) interface, and subject to the analysis methods described in this section.

#### 2.3.1 Antibody-Antigen Interactions

Python package MDAnalysis 0.19.2 was used to identify different types of antibody-antigen and intramolecular antibody interactions, applied to both REST2 trajectories and crystal structures prepared for simulation (61, 62). The interface was defined as residues of any two atoms that are not hydrogens, one from the Fv and one from the antigen, within 4.5 Å of each other. The crystal structure’s interface residue selection was applied to its simulated counterpart’s entire trajectory when making comparisons between them. For comparisons of interface interactions with affinity, all possible interactions between antibody and antigen at the interface were captured as the system fluctuated. For interactions with bulk solvent, where a residue selection was required for the simulation interface, antibody atoms were selected from the NPT equilibrated structure, justified by checks that the interface does not change on a residue basis throughout the REST2 simulation. The interface criteria for these antibody atoms included their hydrogens, as their dynamics are modelled in simulations and we wished to maximize the advantages of *in silico* methods.

Hydrogen bonds were identified using the HydrogenBondAnalysis class, and the subset formed with interface bridging waters was identified using the WaterBridgeAnalysis class. Distance and angle criteria for the different interactions analyzed are given in **Table 2**. Salt bridges were identified using definitions recommended in the MDAnalysis documentation. Interactions with Na^+^ or Cl^-^ ions were defined by applying salt bridge thresholds for any interactions they form with the Fv. Hydrophobic interactions’ distance and angle thresholds, as well as qualifying atom types, were adapted from Arpeggio’s source code and RIP-MD, but these were counted by residue as opposed to individual contacts (63, 64). Intramolecular interaction counts include those with residues i+1, i+2, and i+3.

**Table 2:** Distance and angle criteria for identification of antibody interface interactions.

Interaction type	Criteria
Hydrogen bonds	Heavy atom donor-acceptor distance ≤ 3.5 Å Donor-hydrogen-acceptor angle between 120° and 180°
Salt bridges	Side chain oxygen/nitrogen atom pairs from charged residues Atom pair distance ≤ 4.5 Å
Cation-π interactions	Between positively charged atom and aromatic ring Aromatic ring centroid-charged atom distance ≤ 6 Å Angle between ring centroid-cation vector and normal vector of the ring is either between 0-60° or 120-180°
π-stacking	Distance between two aromatic ring centroids ≤ 6 Å
General hydrophobic contacts	Distance between two hydrophobic atoms ≤ 4.5 Å

#### 2.3.2 CDR Sampling

CDR conformational populations were examined by their extent of dihedral and Cartesian space sampling. Each CDR was measured individually to avoid the noise of other loops obscuring distinct conformations. Dihedral sampling was measured using Dynamics Analysis by Salt and Hudson (DASH), which histograms a time series of each CDR backbone dihedral angle (65). Peaks in the histograms with bins representing at least 1.5% of trajectory frames were combined with the rest of the CDR’s histograms to obtain states. The resulting states that appeared for less than 1% of the trajectory were considered rare and discarded. States that passed these thresholds were compared with each other using DASHSIM, which calculates the circular similarity of two states (66). In brief, differences between the two states’ mean torsion angles are normalized to between 0 and 180°, which are normalized again to give a score between 0 (180° difference) and 1 (0° difference and thus identical). Different states with a circular similarity of 80% or above were considered as the same conformation.

Cartesian space sampling was measured using principal component analysis (PCA) (67). Using GROMACS 2018.2, apo and holo trajectories were superimposed using the backbone of residues making up the V_H_/V_L_ interface. A covariance matrix between the mass-weighted CDR backbone atoms was calculated and diagonalized to generate eigenvectors and their associated eigenvalues. Frames projected along the first three principal components (PCs) were used to identify conformations and quantify the overlap between apo and holo trajectories. Conformations were identified by average linkage hierarchical clustering using the Python package scikit-learn 0.22.1, with a Euclidean linkage distance threshold of 11 (68). This was selected by testing a range of potential thresholds, aiming to minimize differences between clusters identified from the algorithm and those identified by visual inspection. The overlap between apo and holo frames projected along their first three PCs was quantified by MDAnalysis’ dimensionality reduction ensemble similarity (DRES) module (69, 70). Briefly, each trajectory’s projected frames were considered as a representative sample of the CDR’s conformational probability distribution, and the apo and holo trajectories’ probability densities were compared using the Jensen-Shannon divergence.

## 3 Results

In this work, nine antibodies’ Fv-antigen (holo) and Fv only (apo) structures underwent 100 ns of REST2 simulation, and the unscaled replica was analyzed to further our understanding of antibody-antigen interactions. REST2 is an enhanced sampling method that allows more CDR dynamics to be explored than in the same amount of unbiased molecular dynamics. We first outline characteristics of the antibody-antigen interface seen in these trajectories, then describe the behavior of the CDRs, and lastly present correlations from our observations.

### 3.1 Antibody-Antigen Interface

**Figure 1:**
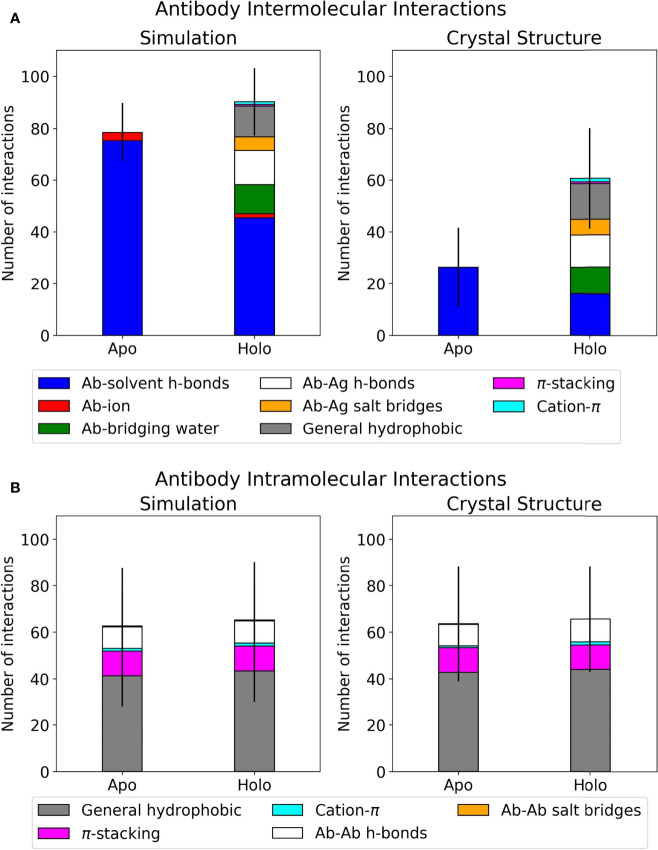
Interface interactions of the antibody-antigen dataset. Error bars show one standard deviation of the mean total. Graphs for individual antibodies are in **Supplementary Figure 1**. **(A)** Mean intermolecular interactions for REST2 simulations and crystal structures. **(B)** Mean intramolecular interactions for REST2 simulations and crystal structures.

Interactions at the antibody-antigen interface were identified using the MDAnalysis Python package, as described in Materials and Methods (61, 62). Each antibody was analyzed in four scenarios: apo crystal structure, holo crystal structure, apo simulation, and holo simulation. The holo crystal structure’s interface residue selection was used across all four scenarios to ensure fair comparisons for interaction counts. Results for the dataset, classified by interaction type, are summarized in **Figure 1**. The counts for direct polar interactions, namely antibody-antigen hydrogen bonds and salt bridges, are similar in simulation and crystal structure. The largest difference between simulated and crystal structure intermolecular interactions lies in the involvement of water. As exemplified in **Figure 1A**, the difference between total polar interactions of apo and holo crystal structures is not seen in simulation, where the discrepancy is filled by interactions with water and a small number of interactions with ions.

When comparing apo and holo simulations (left plot, **Figure 1A**), a decrease in the apo number of hydrogen bonds with water led to an increase in the holo number of direct hydrogen bonds and salt bridges with the antigen; this is unsurprising given that some solvent interactions must be lost and replaced by the antigen. Of these holo interactions, the antibodies formed similar numbers of hydrogen bonds to antigens and bridging waters. Another notable difference in **Figure 1A**’s left plot is the holo bar’s extra hydrophobic interactions, of which there is no apo equivalent. The majority of these are general contacts between hydrophobic atoms. Intramolecular interactions were also analyzed using the same method and are presented in **Figure 1B**. These are very similar between simulation and crystal structure, and the majority of intramolecular interactions involve general hydrophobic contacts. This suggests that changes occurring at the antibody interface upon antigen binding are mostly intermolecular, particularly in interactions with water. We thus present further analyses on the holo antibodies’ hydrogen bonding with water.

**Figure 2:**
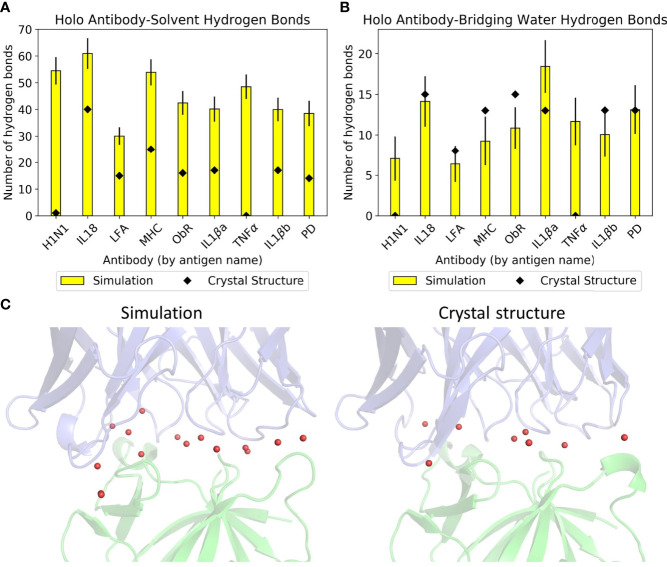
Hydrogen bonding with water in the dataset. Mean numbers of **(A)** antibody-solvent hydrogen bonds and **(B)** antibody-bridging water hydrogen bonds formed by the dataset’s holo complexes, for both their static crystal structures and when simulated. Error bars for the simulation means are given to one standard deviation. **(C)** Anti-IL-1βa’s bridging waters (red spheres) in a representative simulation frame (left) and in the crystal structure (right). The Fv is in blue and the antigen in green.

Simulation of these antibodies in a solvated environment enables a fairer comparison of hydrogen bonding with water across the dataset, as counts from crystal structures alone would depend on the number of solved crystal waters. **Figures 2A, B** show the differences between simulation and crystal structure counts. Some members of the dataset were solved over a decade ago; newer, higher resolution structures that visualize all waters would make our computational results more in line with experimental observations. All antibodies formed more hydrogen bonds with bulk solvent in simulation, and these are most pronounced in anti-H1N1 and anti-TNFα, whose holo structures have one and zero solved crystal waters respectively. The relationship between simulation and crystal structure bridging waters is less consistent, as shown by **Figure 2B** where three antibodies’ bridging waters were underrepresented in simulation (anti-MHC, anti-ObR, anti-IL-1βb) and two were underrepresented in the crystal structure (anti-H1N1, anti-TNFα). Simulation counts of hydrogen bonds with bridging waters also varied more than solvent waters. Of the crystal structures with multiple solved waters, anti-IL-1βa had the largest increase in hydrogen bonding with bridging waters, and an example frame is given in **Figure 2C**. These bridging waters were found across the interface, implying that multiple CDRs involve them in antigen binding.

Given the difficulty in providing crystal structures with sufficient resolution to identify all waters consistently, conclusions on these interfaces were drawn from simulation data. This is further justified by the similar numbers of direct antibody-antigen interactions between simulation and crystal structure. The large amount of hydrogen bonding with water suggests that the antibody-antigen interface is highly water-mediated, an aspect that is not easily captured when examining static crystal structures alone.

### 3.2 CDR Sampling

**Figure 3:**
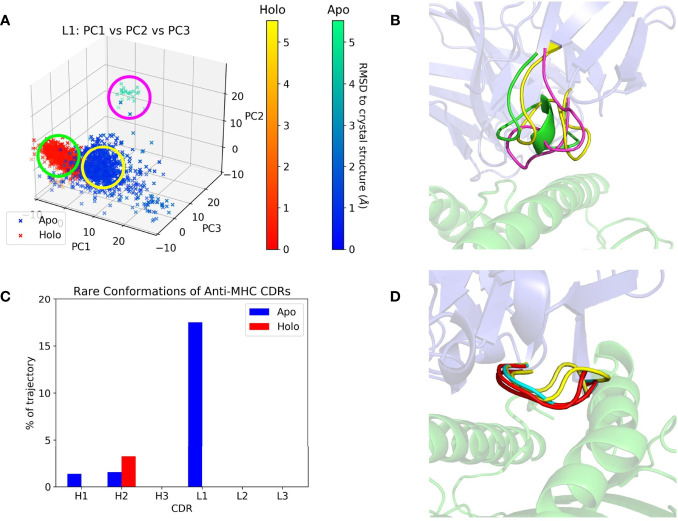
Anti-MHC PCA and DASH examples. The Fv is in blue and the antigen in green on the right subfigures. **(A)** Apo and holo anti-MHC frames projected along the first three PCs of L1, with frames colored by RMSD with respect to their crystal structure conformation. **(B)** Representative structures of L1 conformations, with colors corresponding to the circles in **(A)**. **(C)** Percentage of each CDR’s trajectory classified as rare in DASH, showing H2 with more rare conformations in its holo state. **(D)** Representative structures for H2’s apo-unique (cyan), holo-unique (red), and matched conformations present in both trajectories (yellow).

CDR dynamics during REST2 simulations were measured by their extent of Cartesian space and dihedral angle sampling. Principal component analysis (PCA) of the CDR backbone atoms was used to help visualize the Cartesian coordinate variance. The first three principal components (PCs) from a combined trajectory of apo and holo frames were found to give a sufficient representation, accounting for 60-95% of the variance. Plots of the frames projected along each CDR’s first three PCs are given in **Supplementary Figure 2**, showing that most CDRs did not deviate from the crystal structure throughout the simulation, but apo frames tend to be more spread out than holo ones. The CDR with the highest RMSD relative to its crystal structure, anti-MHC’s L1, is shown in **Figures 3A, B**. Apo L1 clearly sampled more areas of physical space than holo L1, with a cluster of frames having an RMSD with respect to the crystal structure of over 5 Å.

Two methods were then employed to quantify the difference between apo and holo frames projected along their first three PCs, the first being Dimensionality Reduction Ensemble Similarity (DRES) (69). This scores the overlap between apo and holo projections, with 0 signifying identical ensembles and ln (2) signifying no overlap. The mean scores for each CDR across the dataset are plotted in **Supplementary Figure 3A**. Clear overlaps of the standard deviations reflect the large variation in scores, with no CDR having consistently different apo and holo ensembles. The second method involved average linkage hierarchical clustering of these 3D plots, returning numbers of clusters for each CDR’s apo and holo trajectories. Clusters with an average Euclidean linkage distance above 11 were considered separate, and numbers of clusters are given in **Supplementary Table 1**. Apo and holo cluster comparisons are summarized in **Table 3**.

**Table 3:** Comparison of PCA clusters and DASH conformations for each CDR from the dataset.

Method	% of CDRs		
	More apo sampling	Equal sampling	More holo sampling
PCA (Cartesian analysis)	38.9	57.4	3.7
DASH (dihedral analysis)	22.2	75.9	1.9

Percentages are rounded to one decimal place.

PCA describes a CDR’s conformation in each frame as a single point in PC space. It thus only shows large, slow changes in physical space, potentially obscuring any small motions of the CDR. We thus employed Dynamics Analysis by Salt and Hudson (DASH) as a complementary analysis method. Dihedral distributions of the CDRs were plotted as histograms, and states generated by combining peaks in different plots whilst discarding rare conformations. Again, the remaining states were counted and compared, with counts and rare conformation percentages in **Supplementary Tables 2, 3**. Apo and holo states were also matched using circular similarity, allowing an estimate of their conformational overlap. Percentages of matched states are in **Supplementary Table 4**, and apo/holo comparisons are also summarized in **Table 3**. CDRs were either equally or more flexible in their apo form except for anti-MHC’s H2, which had a higher rare conformation percentage and more conformations when antigen bound, as presented in **Figures 3C, D**.

Both Cartesian space and dihedral analysis show that conformational populations of CDRs are not the same upon antigen binding. A higher proportion of CDRs sampled more in their apo state than *vice versa*, suggesting these loops are more flexible in the absence of the antigen. There is little overlap in the PCA clusters when quantified using DRES (**Supplementary Figure 3**), as apo frames tend to be spread wider by the most significant PCs (**Supplementary Figure 2**). However, significant overlap is seen in dihedral analysis when apo and holo were compared using circular similarity, as binding state-specific conformations form a minority of a CDR’s overall dihedral population (**Supplementary Table 4**). We thus conclude that CDRs are more flexible when the antigen is absent, but the difference is more pronounced in Cartesian space analysis than dihedral analysis. In the latter, more flexible apo CDRs visit higher numbers of unique conformations, but these tend to be less populated than the matched ones.

### 3.3 Correlations

Following our observations of hydrogen bonding dominating intermolecular interactions and varying CDR flexibilities in different antibodies, we have yet to answer the question of whether this provides any indication of affinity. Since affinity is not dictated by any single CDR, comparisons were made to metrics that included all six CDRs of an antibody, quantified by squaring the Pearson correlation coefficient (R^2^). Our first interesting observation is the lack of correlation between an antibody’s total CDR length or molecular weight with its affinity (**Supplementary Figures 4A, B**), showing that the size of CDRs do not dictate their binding. In terms of flexibility, we used each antibody’s mean DASH and PCA cluster counts. Comparisons of individual CDR lengths against our clustering results are in **Supplementary Figure 5**, confirming that longer CDRs are not always more flexible. Their length alone did not provide detailed information on their behavior.

**Figure 4:**
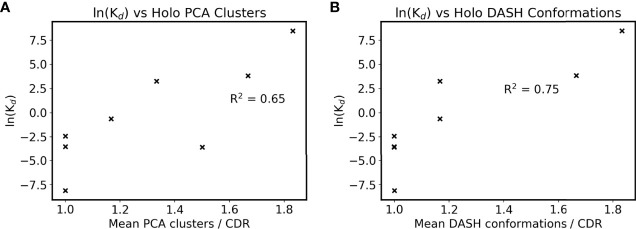
Antigen bound CDR sampling and affinity. The mean number of **(A)** holo PCA clusters and **(B)** holo DASH conformations across all six CDRs is plotted for each antibody. Note that two points overlap in subfigure B at ln(K_d_) = -3.5. ln(K_d_) is used as a proxy for affinity due to the Gibbs free energy equation of ΔG° = -RTln(K_eq_), and natural logarithms of K_d_ values in nM are used (see **Table 1**).

Clustering results were then compared to their SPR-determined affinities as listed in **Table 1**, with the exception of anti-ObR which only had IC_50_ data. The antibodies’ mean DRES scores, a measure of the overlap between apo and holo frames when projected along their first three PCs, are plotted against affinity in **Supplementary Figure 3B**. No correlation is present between ensemble similarity and affinity, with an R^2^ of 0.28. The quantified flexibility of apo and holo CDRs were next examined separately, by plotting affinity against mean PCA cluster and DASH conformation counts. There is no discernible correlation between apo counts and affinity (**Supplementary Figures 4C, D**). However, an arguably positive correlation is seen for holo counts in **Figures 4A, B**. When the difference between apo and holo clustering is plotted in **Supplementary Figures 4E, F**, this trend disappears. Instead, the numerically small differences between apo and holo suggest that some CDRs are preorganized for antigen binding. Together, these correlations further support that CDR flexibility after the formation of antibody-antigen interactions may be linked to binding affinity.

**Figure 5:**
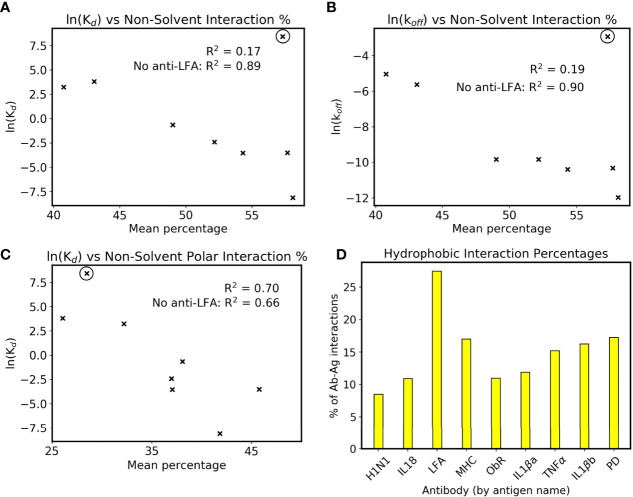
Interactions and affinity. The anti-LFA outlier is marked with a black circle, and natural logarithms of K_d_ values in nM are used (see **Table 1**). **(A)** ln(K_d_) and **(B)** ln(k_off_) are plotted against the percentage of intermolecular interactions that are not formed with bulk solvent. **(C)** ln(K_d_) against the percentage of intermolecular interactions that are neither bulk solvent nor hydrophobic contacts. **(D)** Mean percentage of intermolecular interactions that are hydrophobic in the dataset’s simulated holo complexes.

Interface interactions from the holo simulations were explored further to rationalize this link. When comparing simulations to crystal structures in **Figure 1**, the latter’s interface residue selection was used in all trajectory frames to allow a fair comparison. For identifying trends with affinity, we wished to account for the dynamics of the system, and all possible interactions with the antigen were counted to fully capture the antibody’s behavior. The resulting mean counts for each intermolecular interaction type are plotted against antibody affinities in **Supplementary Figure 6**. The interactions show little correlation with affinity when plotted individually, except for the number of hydrogen bonds with bulk solvent in **Supplementary Figure 6A**. To make this more applicable to affinity prediction, intermolecular interactions not to bulk solvent are plotted as a percentage of all intermolecular interactions in **Figure 5A**. Bridging waters are included as an intermolecular interaction as they indirectly form an antibody-antigen contact. Although the R^2^ of the eight antibodies is only 0.17, omission of anti-LFA’s count increases this to 0.89, demonstrating a positive correlation in the remaining eight. Further inspection showed that the correlation with K_d_ is more associated with the reported k_off_ values (**Figure 5B**). Combined with the observation that the change in CDR flexibility is not correlated with binding affinity, which could imply a more entropic component, formation of more antibody-antigen interactions in higher affinity antibodies suggest that antibody binding is more enthalpy-driven, although solvent-mediated entropic effects cannot be ruled out.

We have however identified that anti-LFA is different from the rest of the dataset. Firstly, it is the only antibody where a non-solvent ion is part of the epitope. Secondly, it has an experimental affinity of 4.7 µM, a 100-fold increase from the next lowest affinity of anti-MHC’s 46 nM, and the SPR was done using Mg^2+^ buffer as opposed to the crystal structure’s Mn^2+^ (32, 33). An attempt was made to include anti-LFA by plotting the non-solvent polar intermolecular interaction percentage, i.e. not bulk solvent and not hydrophobic interactions, and this gives a slightly weaker R^2^ of 0.70 (**Figure 5C**). The interactions measured using fluctuating simulation interfaces further reveal that anti-LFA forms proportionally more hydrophobic interactions than the rest of the dataset (**Figure 5D**). We thus propose that anti-LFA’s interface may not be representative of a typical antibody, and affinity is more likely related to the number of specific antibody-antigen interactions. From the remaining seven, an increased proportion of non-solvent interactions with the antigen may be enabling the increased rigidity of antigen bound CDRs.

## 4 Discussion

In this work, we simulated nine antibody-antigen crystal structure pairs, enhancing sampling of CDRs due to their large role in dictating binding. By studying the types of interactions formed, we identified that the antibody-antigen interface had large numbers of waters. Compared to crystal structures, simulated complexes tend to form more hydrogen bonds with water and fewer hydrophobic contacts. However, the work presented here only specifically increased CDR sampling, meaning that bridging water interactions were not necessarily explored as extensively. The presence of water at these interfaces, and their potential role in facilitating interactions, has been identified in antibody crystal structures since the 1990s (71, 72). A more recent study on crystal structures of 145 protein-protein interfaces also concluded that when compared to non-antibody complexes, a mean of eleven more hydrogen bonds with waters are found at antibody-antigen interfaces (73). These results on a dataset larger than ours support the idea that water-mediated antibody-antigen interactions are important.

Further inspection of these interfaces revealed that higher affinity antibodies had a higher percentage of non-solvent interactions with their antigen (**Figure 5A**). This was only present when defined interfaces fluctuated with the simulation, as the equivalent plot using crystal structure interfaces showed a lower correlation with affinity (**Supplementary Figure 7**). Firstly, this demonstrates how simulation of antibody dynamics can provide new information for these interfaces. Secondly, it suggests that water has an important role at the interface, as ‘non-solvent interactions’ included hydrogen bonds to bridging waters. However, as previously mentioned, current computational design methods do not measure these interactions, as they rely on implicit solvation models. Given **Figure 5A**’s high R^2^ of 0.89 when excluding the anti-LFA anomaly, we suggest that explicit solvent would better capture antibody-antigen interactions *in silico*.

We have also found that CDRs behave differently in the presence of their antigen. Over half were equally flexible in antigen bound and unbound simulations, suggesting their preorganization for antigen binding. This is in line with our observation of higher affinity antibodies having less flexible antigen bound CDRs, with R^2^s of 0.75 and 0.65 when dihedral and Cartesian clustering were respectively plotted against affinity. Preorganization has been proposed as a method to reduce cross-reactivity (74–77), but this may be exaggerated (78). This exaggeration could explain the poor correlation between affinity and the apo/holo overlap of an antibody’s CDR conformational population, as well as the CDRs where flexibility changes upon antigen binding. If preorganization were important, a smaller difference between antigen bound and unbound sampling would be correlated with better affinity. The lack of preorganization in some CDRs also has implications for scoring functions that assume identical CDR conformations when antigen bound and unbound; Liu et al. for example measured binding by the difference in system energy between bound and unbound antibody designs (13). This definition would not capture differences in binding state-specific flexibility and could be another reason for the presence of false positives.

We also note that plotting affinity against the differences between apo and holo sampling give much lower R^2^s (**Supplementary Figures 4E, F**). We thus cannot relate antibody affinity to entropically driven changes in CDR flexibility. This does not exclude the possibility of entropic contributions by solvent. Numerous studies have dissected the thermodynamic signature of antibody binding using isothermal titration calorimetry (ITC). The majority propose that antibody binding is predominantly enthalpic (79–81), but two cases of entropy driven binding were also reported. The first involved an antibody against a staphylococcal nuclease-derived peptide, where binding to the peptide was enthalpic but binding to the whole nuclease was entropic (82). However, crystal structures suggested that the enzyme undergoes conformational change to achieve the peptide fragment’s antibody interactions, thereby making binding more entropically driven. The second case did perform ITC against its intended antigen, and the nanobody with the longest and most hydrophobic CDR3 employed entropy driven binding (83). Here, entropic binding could be attributed to antibody flexibility or solvent by the hydrophobic effect.

The correlations presented are not always clear, such as the R^2^ of 0.65 for numbers of holo PCA clusters against affinity. These poorer correlations could be attributed to limitations in our study. The majority of these were imposed by computational limits: a small dataset of only nine antibodies, simulating only the Fv region, and only 100 ns of REST2 simulation per structure. Firstly, we imposed a number of strict criteria to obtain a high quality dataset with a range of affinities, minimal redundancy in antibody and antigen sequences, sufficiently high resolution structures for simulation, no missing Fv residues, and both antigen bound and unbound structures available. For our choice of only simulating the Fv, our current setup already gave very large systems, and involved 40 µs of advanced molecular dynamics simulations using national level supercomputing services, not including preliminary work to optimize our protocols. These large systems and usage of the REST2 technique heavily limited simulation lengths, and in certain cases the conformational populations of CDRs have not converged (**Supplementary Figure 8**). Additionally, the modelling of anti-LFA was suboptimal, due to a Mn^2+^ ion present in the epitope. Simulation of metal ions by point charge models has long posed many challenges, and in the absence of quantum mechanics methods, the results regarding that structure may be less accurate than the rest of the dataset (84). We acknowledge that this study, despite our best efforts, only offers a limited insight into these complexes. Despite these limitations, the dataset presented here is the longest timescale simulation of antibodies to our knowledge. Here we correlate antibody dynamics with measured binding affinities, and the conclusions that higher affinity antibodies are more rigid and form more antigen interactions are not unexpected.

To conclude, we have analyzed the antibody-antigen interface using extensive simulations of nine antibody-antigen crystal structure pairs. The analysis confirms that antibodies regularly interact with waters at the interface, a feature only captured by high resolution crystal structures. Reduced interactions with solvent waters may facilitate rigidity of CDRs in antibody-antigen complexes, and these interlinked features could provide some indication of an antibody’s binding affinity. Despite their importance, current protocols’ usage of implicit water models omits such interactions in affinity prediction methods. We propose that incorporation of explicit waters may help improve scoring of rationally designed antibodies.

We have also computationally demonstrated enthalpy driven binding *via* analysis of CDR conformations. Using two different metrics to measure CDR flexibility, we showed that some loops behave differently when antigen bound or unbound. Furthermore, differences in CDR flexibility in the presence or absence of the antigen had no correlation with affinity. Instead, some CDRs were preorganized for binding, and affinity was somewhat related to antigen bound sampling. Our calculations seem to confirm the observations by others that antibody binding is enthalpy-driven rather than entropy-driven, and that CDR flexibility should be considered in computational design methods.

Longer simulations combined with further annotation of flexibility and interface interactions would provide a clearer picture of antibody-antigen interactions in the future. We anticipate this would allow better prediction of crucial residues for binding and how their mutation affects affinity. Deeper understanding of these interfaces will enable computational methods to better complement generating antibodies experimentally, as well as improve the current state of the art of rational design against specific epitopes.

## Data Availability Statement

The raw data supporting the conclusions of this article will be made available by the authors, without undue reservation.

## Author Contributions

SK, XL, RT, TB, and JE designed the research. MW performed the research. MW, SK, XL, RT, TB, and JE analyzed and interpreted the data. MW wrote the first draft of the manuscript. MW, SK, RT, TB, and JE contributed to manuscript revision, read, and approved the submitted version.

## Funding

MW thanks the Biotechnology and Biological Sciences Research Council (grant no. BB/P504713/1) and Union Chimique Belge for funding. The ARCHER computing resource was supported by the Engineering and Physical Sciences Research Council (grant no. EP/R029407/1).

## Conflict of Interest

MW’s studentship was partially funded by UCB. MW, SK, and RT are employees of UCB. XL and TB were former employees of UCB, and XL is a current employee of AngitiaBio. JE’s research is partially funded by UCB.

## Publisher’s Note

All claims expressed in this article are solely those of the authors and do not necessarily represent those of their affiliated organizations, or those of the publisher, the editors and the reviewers. Any product that may be evaluated in this article, or claim that may be made by its manufacturer, is not guaranteed or endorsed by the publisher.
