# Corrigendum: Higher Affinity Antibodies Bind With Lower Hydration and Flexibility in Large Scale Simulations

**DOI:** 10.3389/fimmu.2022.969176

**Published:** 2022-07-04

**Authors:** Mabel T. Y. Wong, Sebastian Kelm, Xiaofeng Liu, Richard D. Taylor, Terry Baker, Jonathan W. Essex

**Affiliations:** ^1^ School of Chemistry, University of Southampton, Southampton, United Kingdom; ^2^ UCB, Slough, United Kingdom

**Keywords:** antibody-antigen interactions, antibody binding, antibody affinity, antibody interface hydration, CDR flexibility, molecular dynamics, replica exchange

In the original article, there was an error in affiliations 2 and 3. Instead of “Computer Aided Drug Design, Union Chimique Belge (UCB) Celltech, Slough, United Kingdom” and “Structural Biology, Union Chimique Belge (UCB) Celltech, Slough, United Kingdom”, respectively, they should both be corrected to “UCB, Slough, United Kingdom”.

The authors apologize for this error and state that this does not change the scientific conclusions of the article in any way. The original article has been updated.

## Publisher’s Note

All claims expressed in this article are solely those of the authors and do not necessarily represent those of their affiliated organizations, or those of the publisher, the editors and the reviewers. Any product that may be evaluated in this article, or claim that may be made by its manufacturer, is not guaranteed or endorsed by the publisher.

