# Supplementary material for: Higher Affinity Antibodies Bind With Lower Hydration and Flexibility in Large Scale Simulations
Source: Front Immunol. 2022 May 30;13:884110. doi: 10.3389/fimmu.2022.884110 (PMC9190259; doi:10.3389/fimmu.2022.884110)
Supplement: Supplementary file 1 [file DataSheet_1.pdf]

Supplementary Material

1 Supplementary Figures

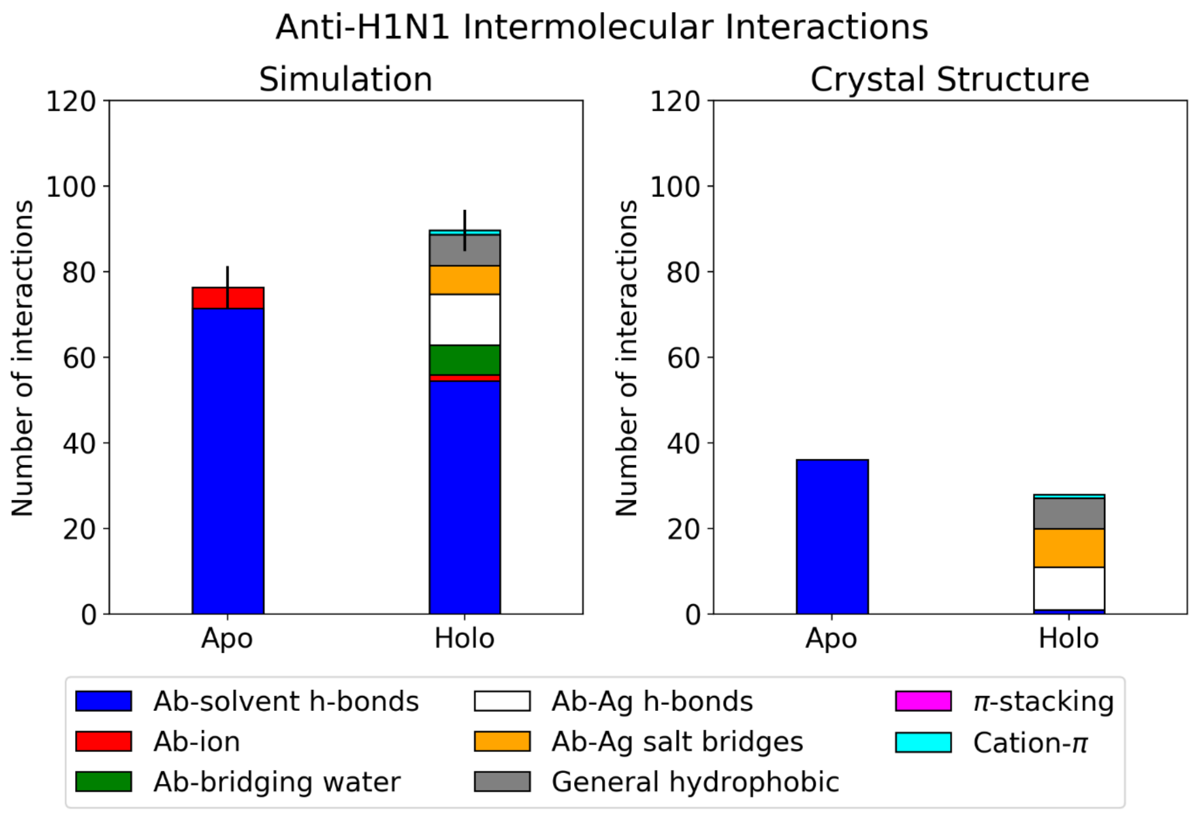

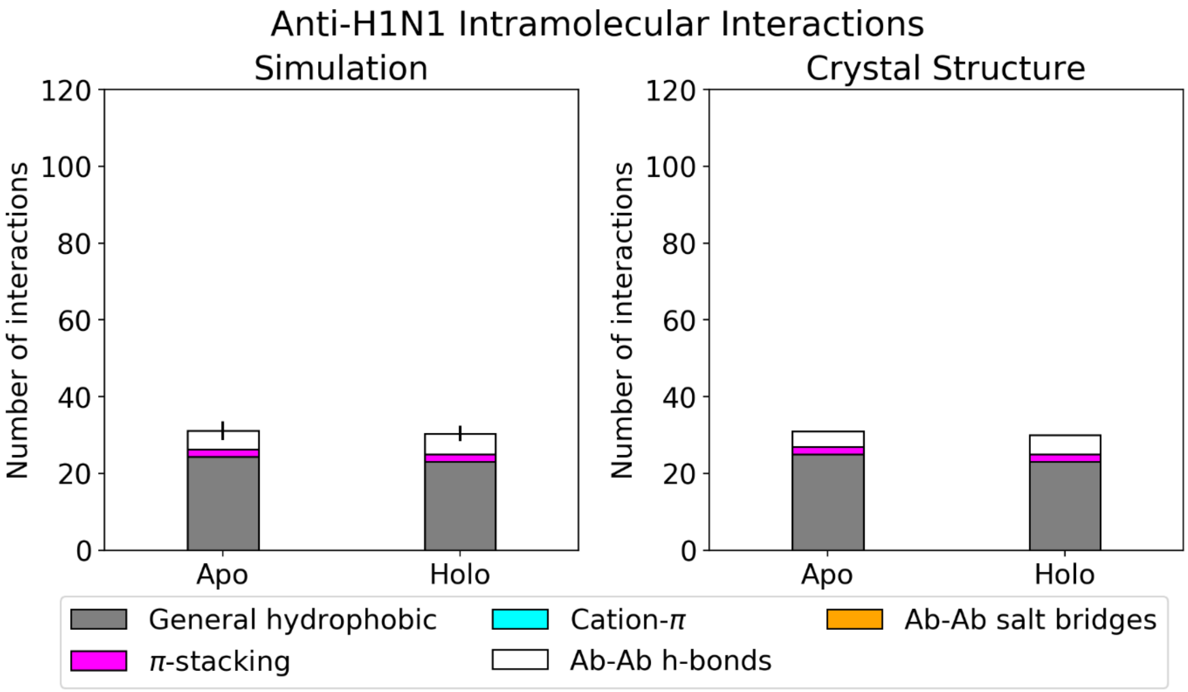
Anti-H1N1 Intramolecular Interactions
Simulation
Crystal Structure
Number of interactions
0
20
40
60
80
100
120
Apo
Holo
General hydrophobic
π-stacking
Cation-π
Ab-Ab h-bonds
Ab-Ab salt bridges

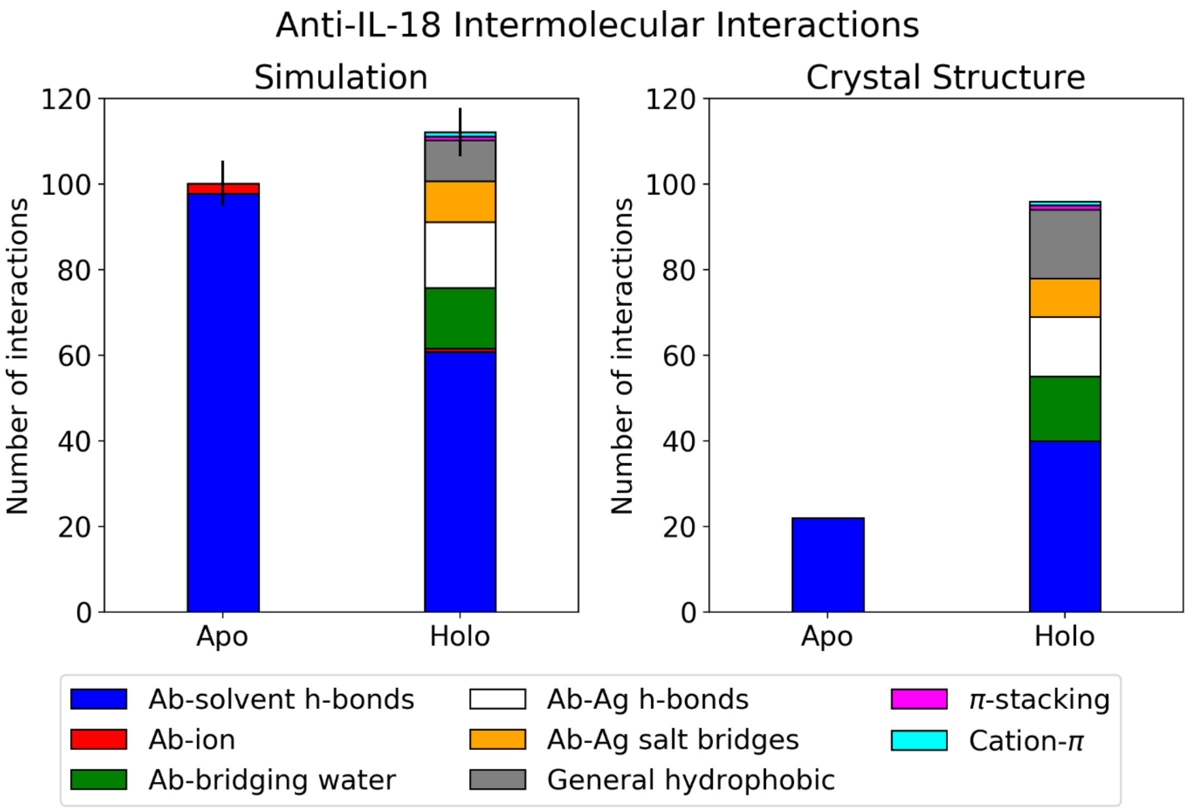
Anti-IL-18 Intermolecular Interactions
Simulation
Crystal Structure
Number of interactions
0
20
40
60
80
100
120
Apo
Holo
Ab-solvent h-bonds
Ab-ion
Ab-bridging water
Ab-Ag h-bonds
Ab-Ag salt bridges
General hydrophobic
π-stacking
Cation-π

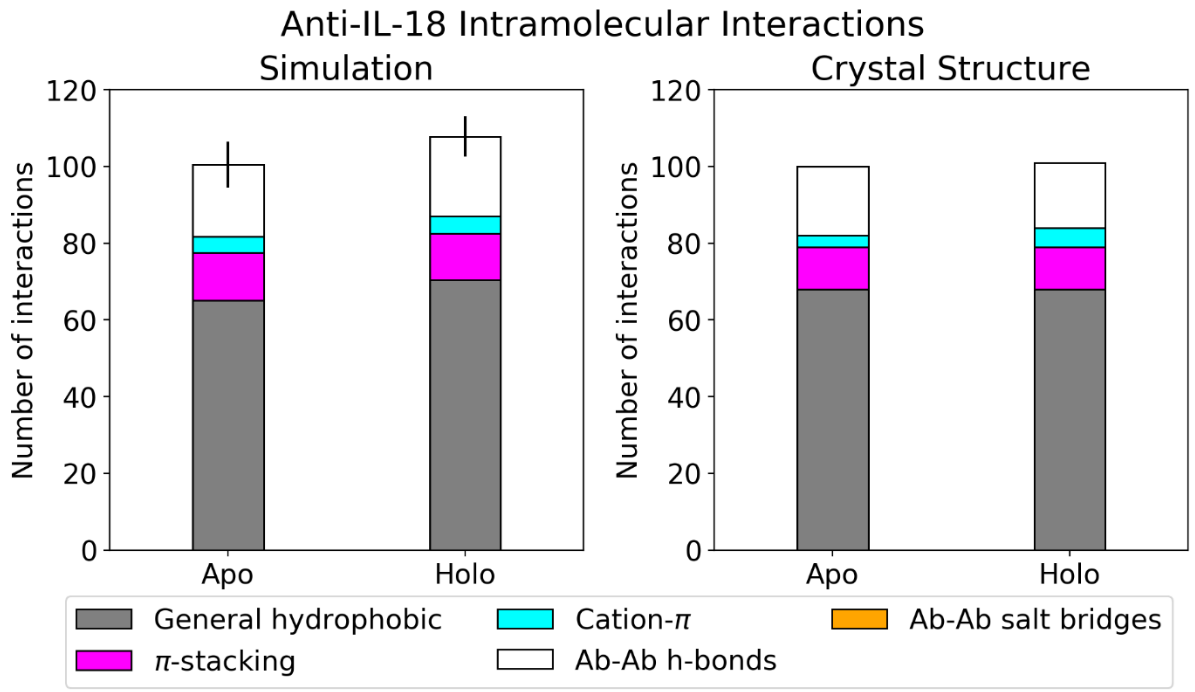
Anti-IL-18 Intramolecular Interactions
Simulation
Crystal Structure
Number of interactions
Apo
Holo
General hydrophobic
Cation-π
Ab-Ab salt bridges
π-stacking
Ab-Ab h-bonds

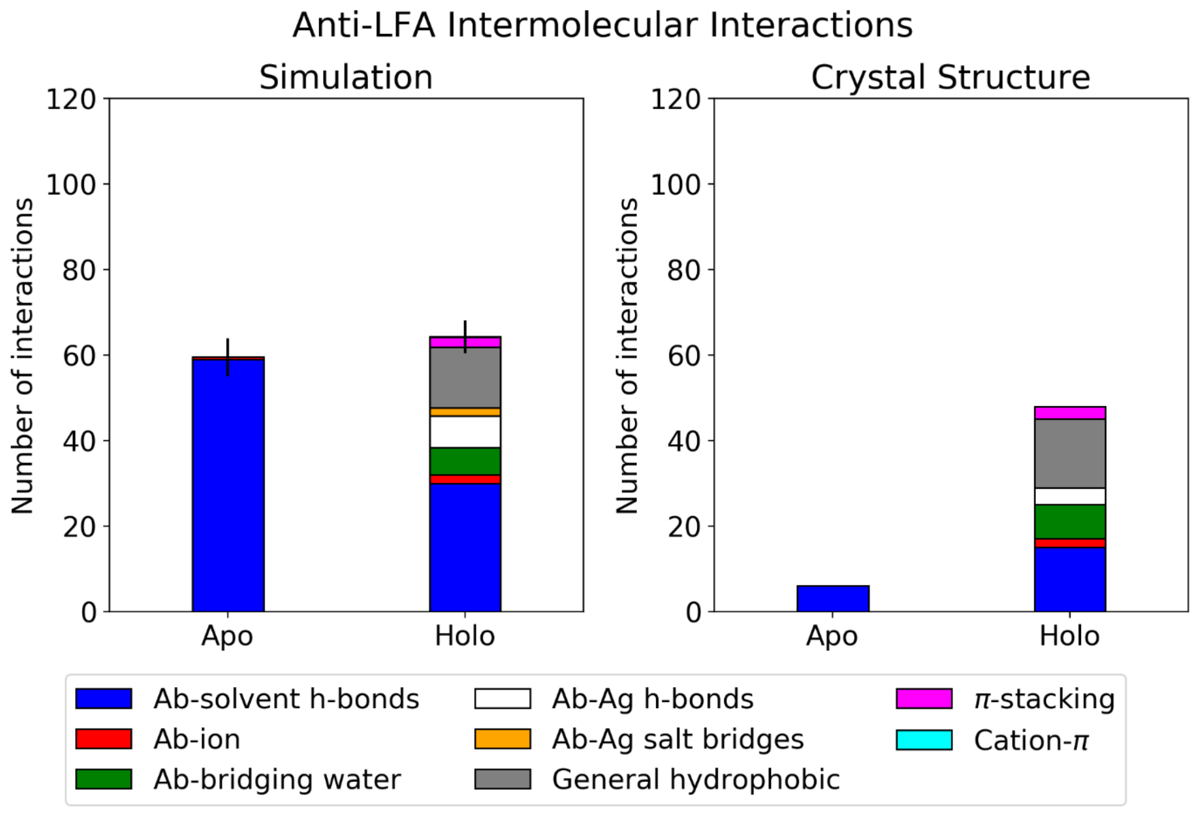
Anti-LFA Intermolecular Interactions
Simulation
Crystal Structure
Number of interactions
Apo
Holo
Ab-solvent h-bonds
Ab-Ag h-bonds
π-stacking
Ab-ion
Ab-Ag salt bridges
Cation-π
Ab-bridging water
General hydrophobic

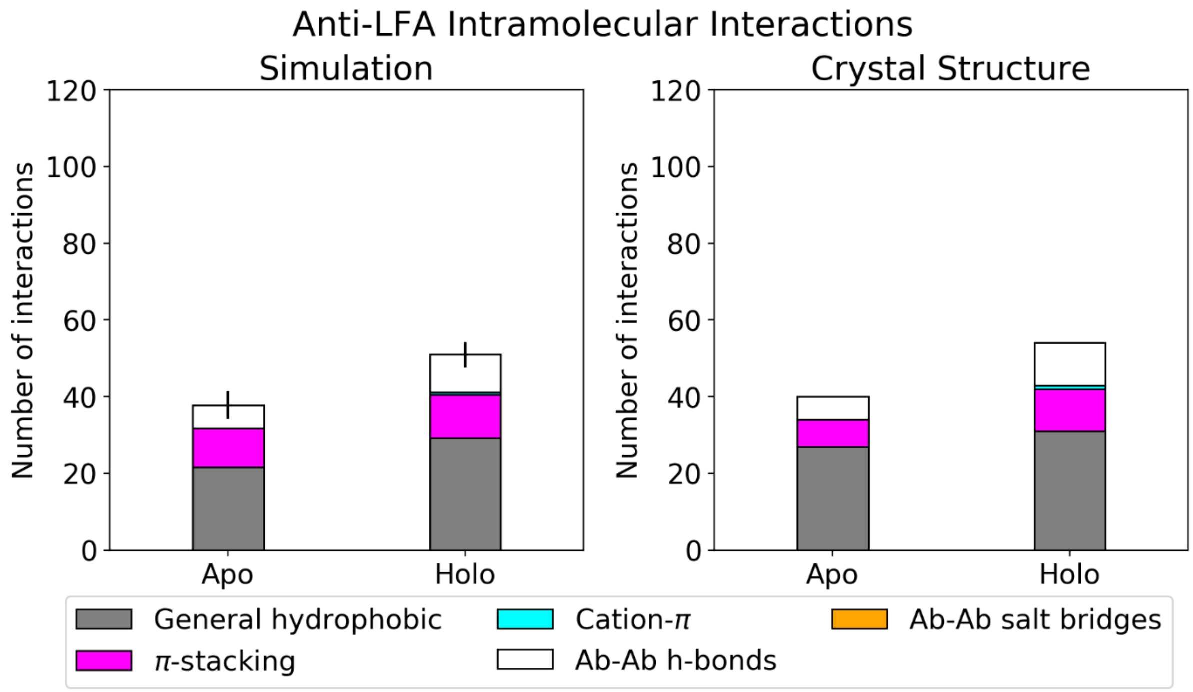
Anti-LFA Intramolecular Interactions
Simulation
Crystal Structure
Number of interactions
0
20
40
60
80
100
120
Apo
Holo
General hydrophobic
π-stacking
Cation-π
Ab-Ab h-bonds
Ab-Ab salt bridges

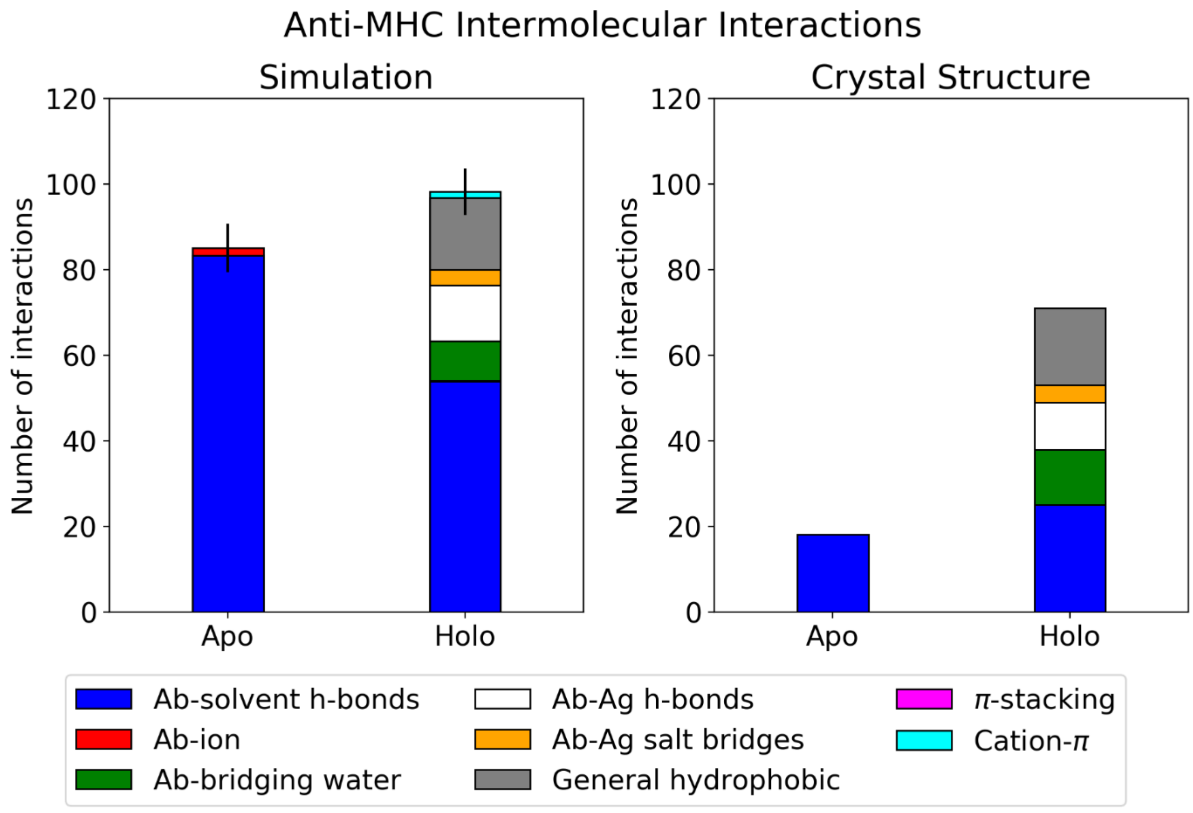
Anti-MHC Intermolecular Interactions
Simulation
Crystal Structure
Number of interactions
0
20
40
60
80
100
120
Apo
Holo
Ab-solvent h-bonds
Ab-ion
Ab-bridging water
Ab-Ag h-bonds
Ab-Ag salt bridges
General hydrophobic
π-stacking
Cation-π

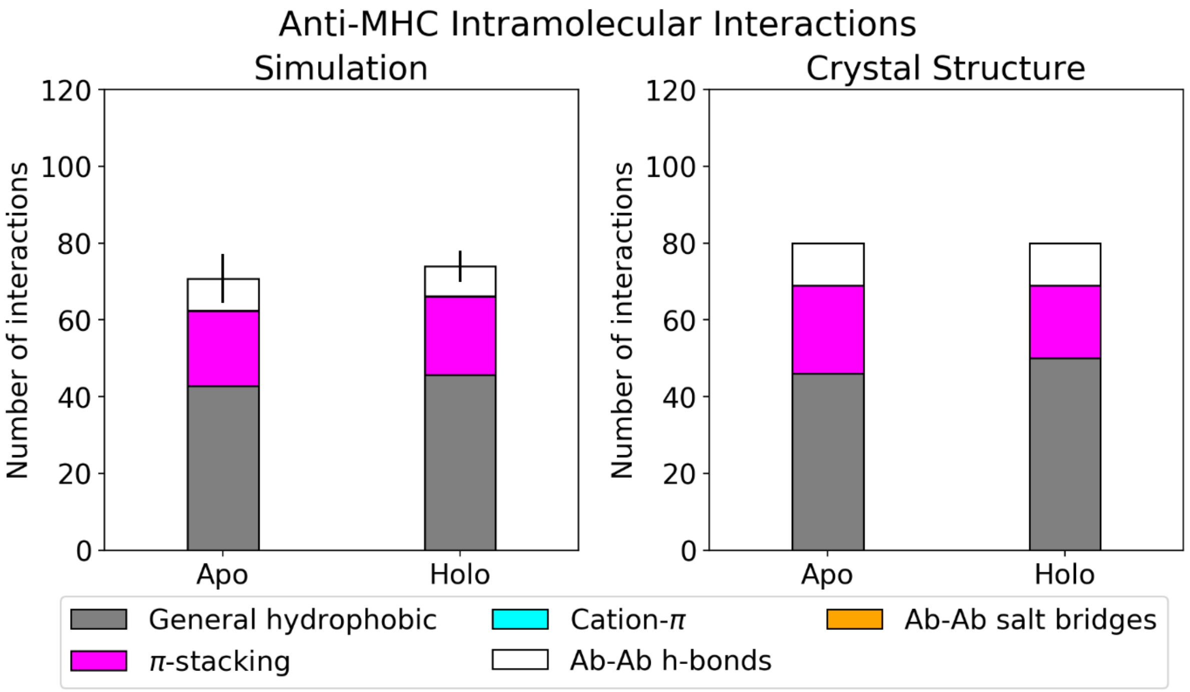
Anti-MHC Intramolecular Interactions
Simulation
Crystal Structure
Number of interactions
Apo
Holo
General hydrophobic
Cation-π
Ab-Ab salt bridges
π-stacking
Ab-Ab h-bonds

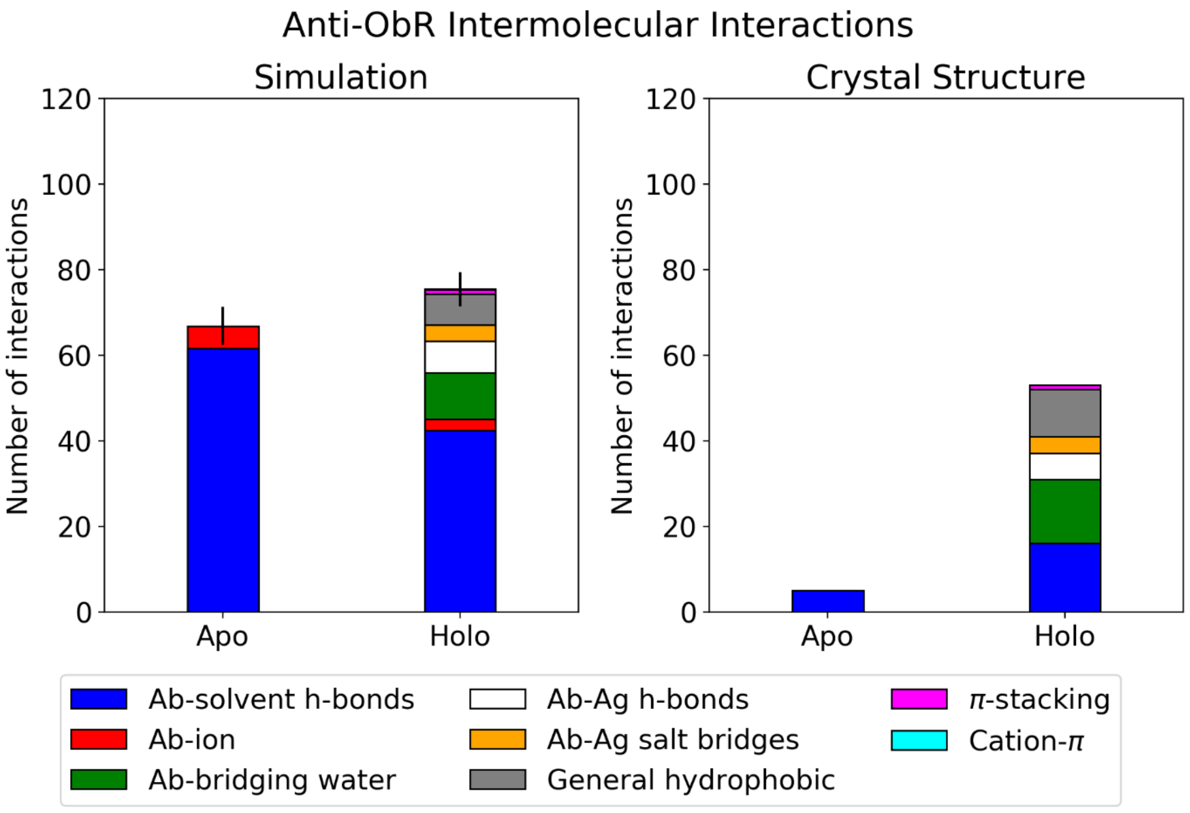
Anti-ObR Intermolecular Interactions
Simulation
Crystal Structure
Number of interactions
Apo
Holo
Ab-solvent h-bonds
Ab-Ag h-bonds
π-stacking
Ab-ion
Ab-Ag salt bridges
Cation-π
Ab-bridging water
General hydrophobic

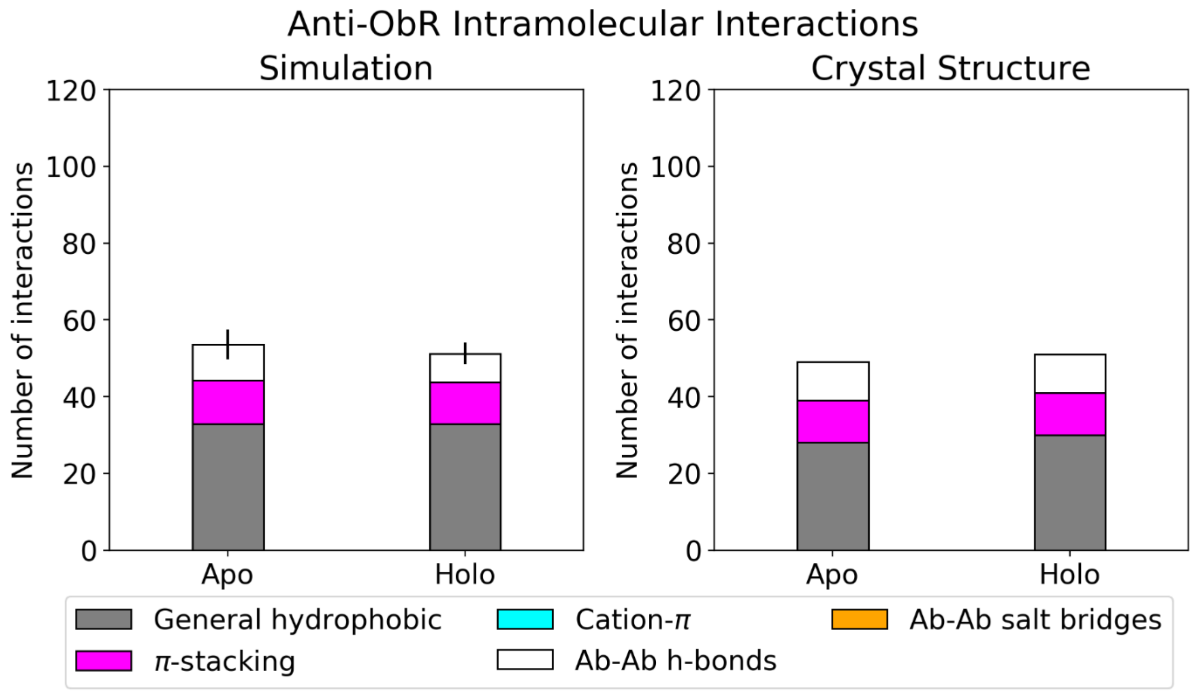
Anti-ObR Intramolecular Interactions
Simulation
Crystal Structure
Number of interactions
0
20
40
60
80
100
120
Apo
Holo
General hydrophobic
π-stacking
Cation-π
Ab-Ab h-bonds
Ab-Ab salt bridges

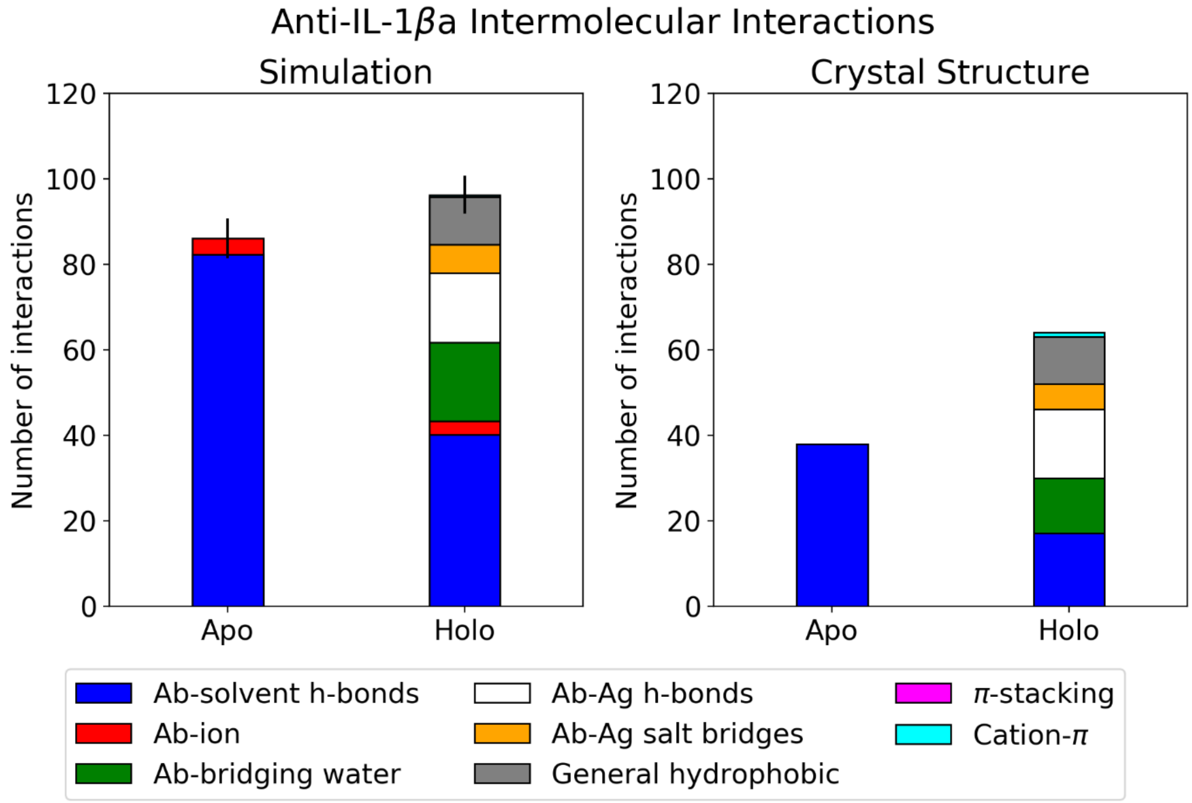
Anti-IL-1βa Intermolecular Interactions
Simulation
Crystal Structure
Number of interactions
0
20
40
60
80
100
120
Apo
Holo
Ab-solvent h-bonds
Ab-ion
Ab-bridging water
Ab-Ag h-bonds
Ab-Ag salt bridges
General hydrophobic
π-stacking
Cation-π

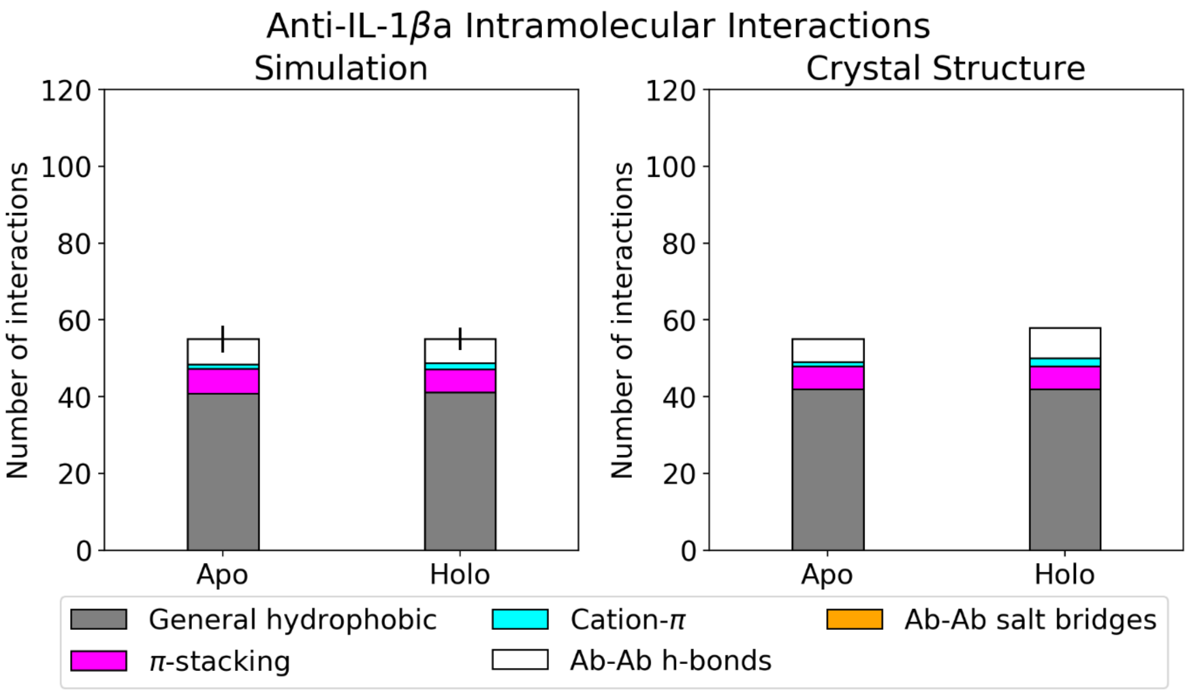
Anti-IL-1βa Intramolecular Interactions
Simulation
Crystal Structure
Number of interactions
0
20
40
60
80
100
120
Apo
Holo
General hydrophobic
π-stacking
Cation-π
Ab-Ab h-bonds
Ab-Ab salt bridges

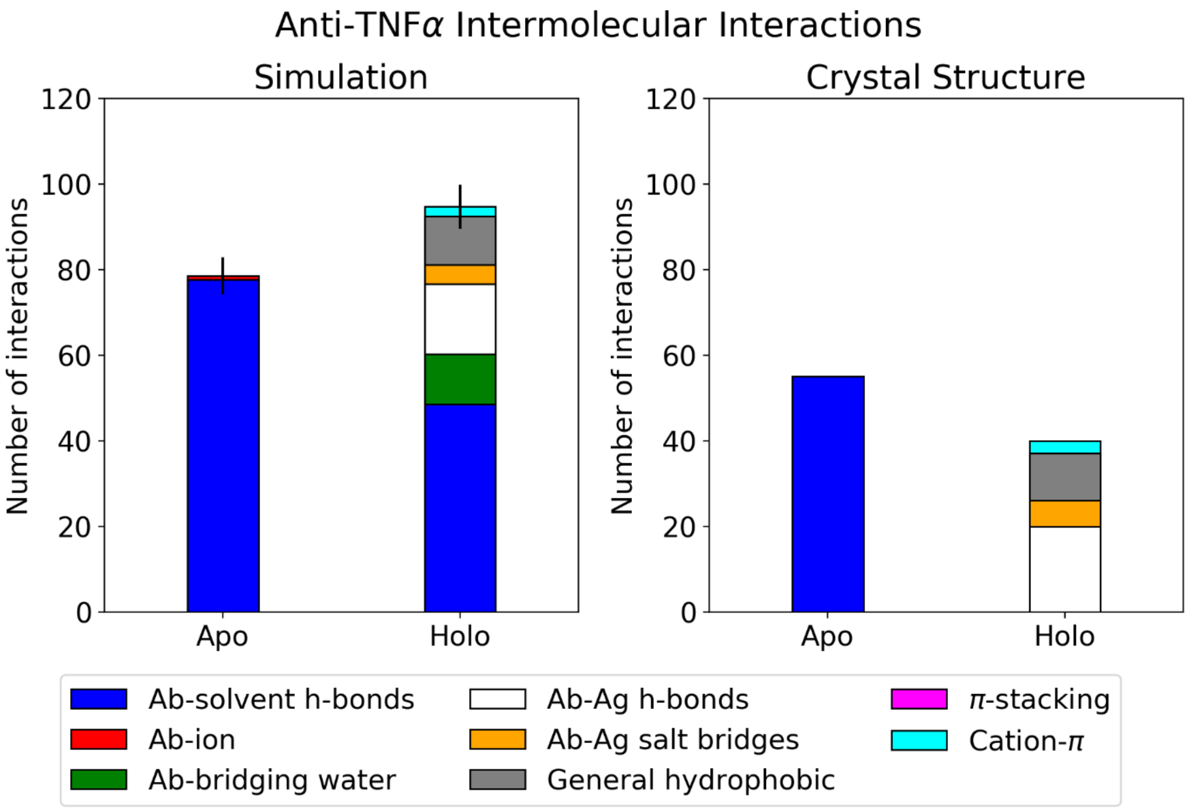
Anti-TNFα Intermolecular Interactions
Simulation
Crystal Structure
Number of interactions
0
20
40
60
80
100
120
Apo
Holo
Ab-solvent h-bonds
Ab-ion
Ab-bridging water
Ab-Ag h-bonds
Ab-Ag salt bridges
General hydrophobic
π-stacking
Cation-π

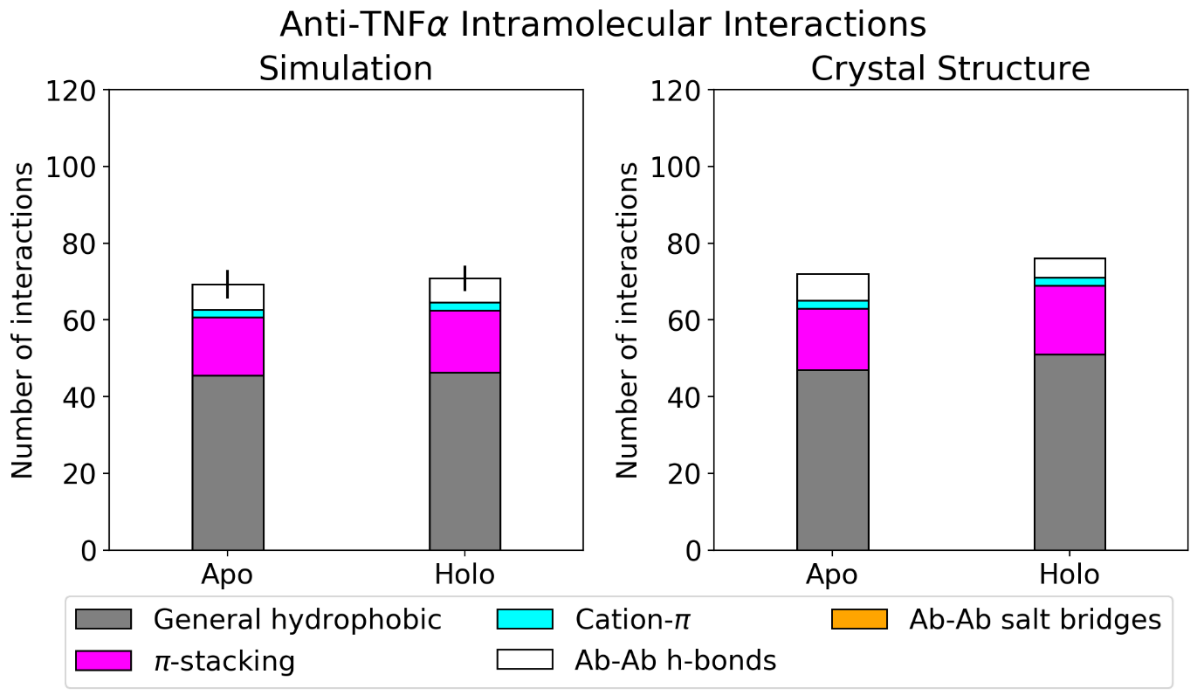
Anti-TNFα Intramolecular Interactions
Simulation
Crystal Structure
Number of interactions
0
20
40
60
80
100
120
Apo
Holo
General hydrophobic
π-stacking
Cation-π
Ab-Ab h-bonds
Ab-Ab salt bridges

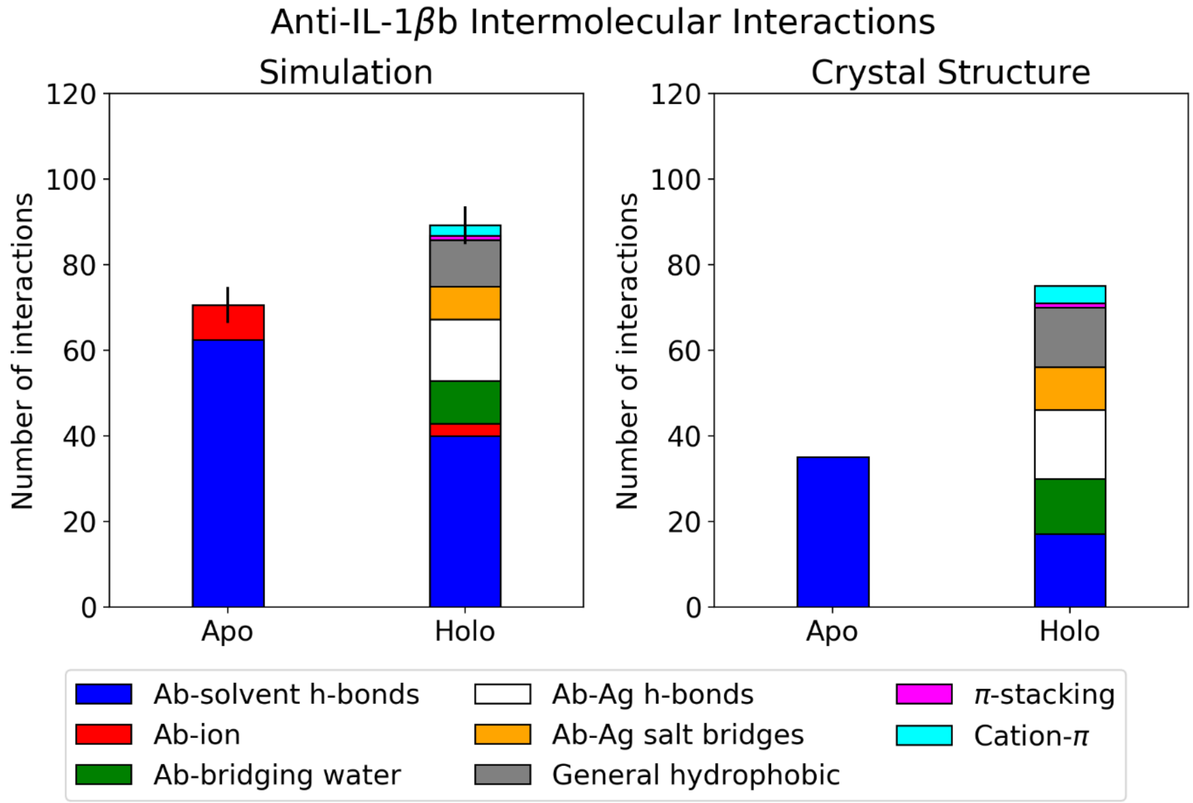
Anti-IL-1βb Intermolecular Interactions
Simulation
Crystal Structure
Number of interactions
0
20
40
60
80
100
120
Apo
Holo
Ab-solvent h-bonds
Ab-ion
Ab-bridging water
Ab-Ag h-bonds
Ab-Ag salt bridges
General hydrophobic
π-stacking
Cation-π

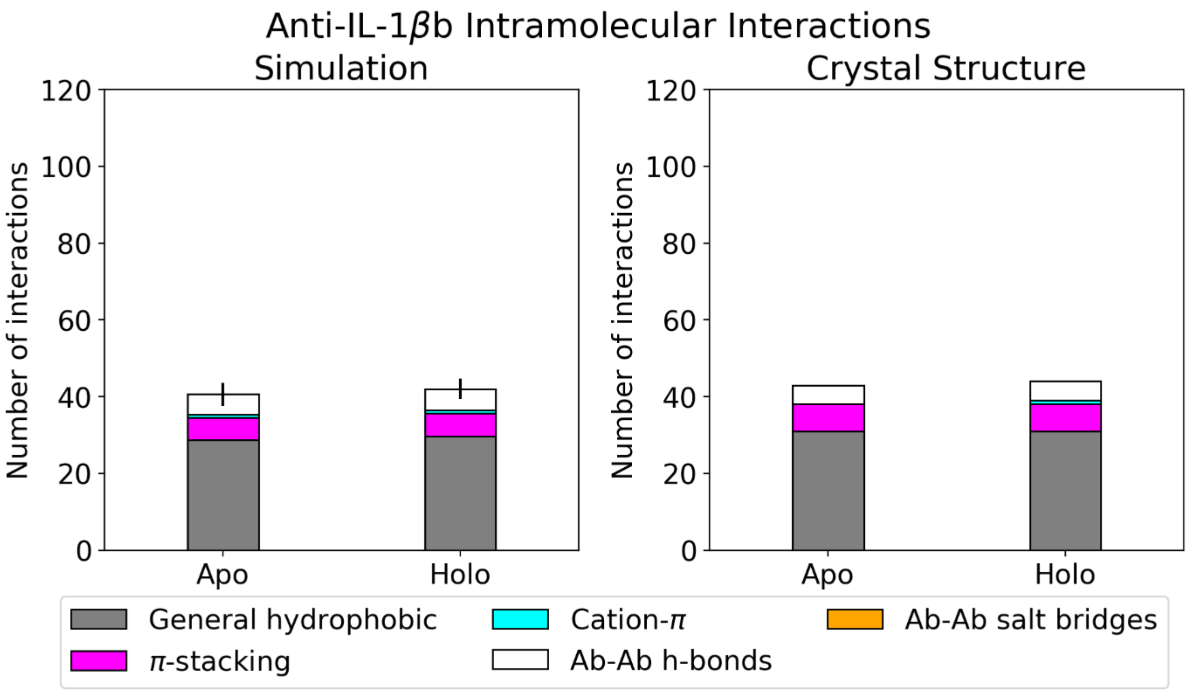
Anti-IL-1βb Intramolecular Interactions
Simulation
Crystal Structure
Number of interactions
0
20
40
60
80
100
120
Apo
Holo
General hydrophobic
π-stacking
Cation-π
Ab-Ab h-bonds
Ab-Ab salt bridges

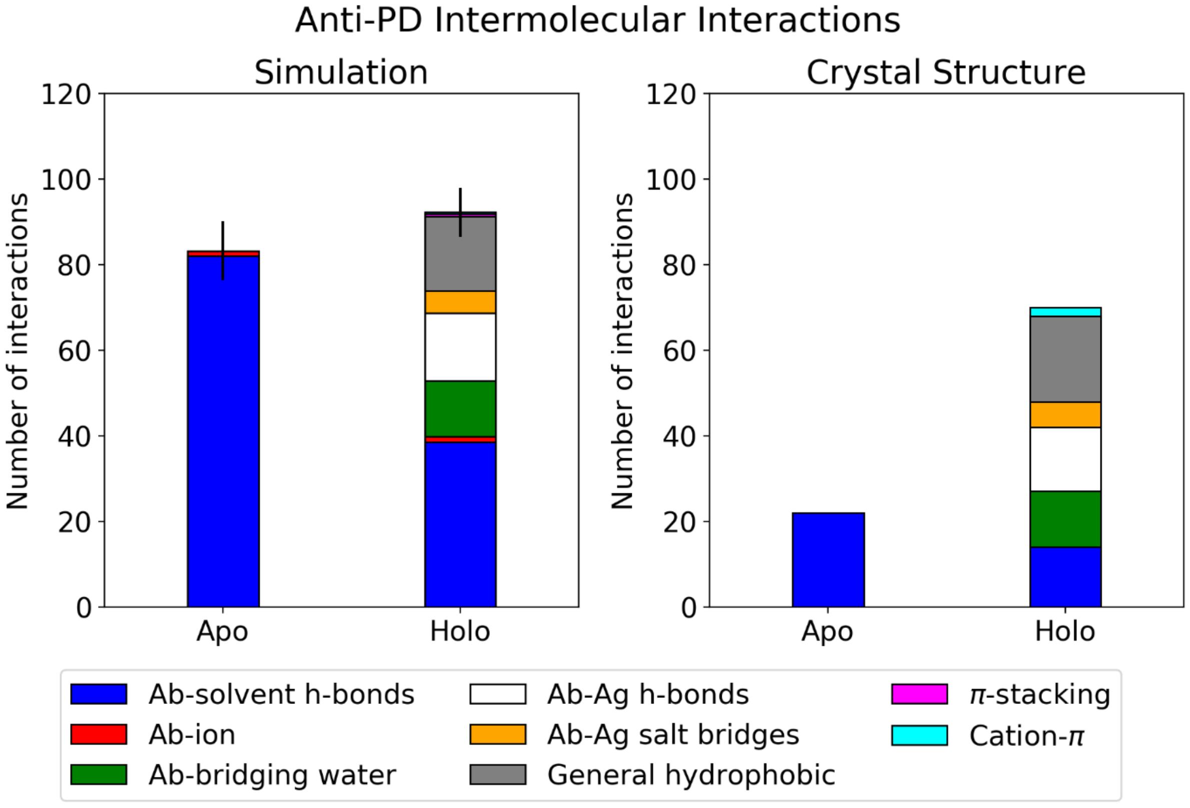
Anti-PD Intermolecular Interactions
Simulation
Crystal Structure
Number of interactions
0
20
40
60
80
100
120
Apo
Holo
Ab-solvent h-bonds
Ab-ion
Ab-bridging water
Ab-Ag h-bonds
Ab-Ag salt bridges
General hydrophobic
π-stacking
Cation-π

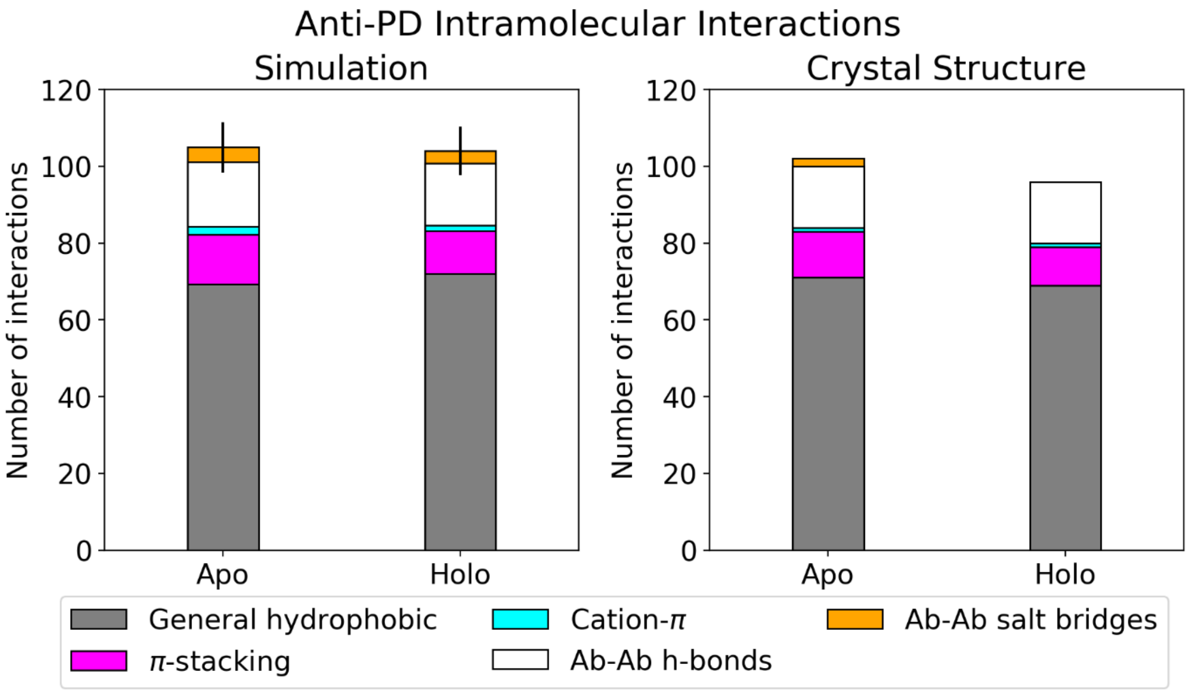

Supplementary Figure 1. Interface interaction plots for the antibody-antigen dataset. Error bars on the left plots represent one standard deviation of the total intermolecular or intramolecular interactions formed throughout the REST2 simulation. Crystal structure values for interactions with ions are zero as the solvent is added during simulation setup. The exception is anti-LFA, where the epitope involves a metalloprotein's ion.

Anti-H1N1

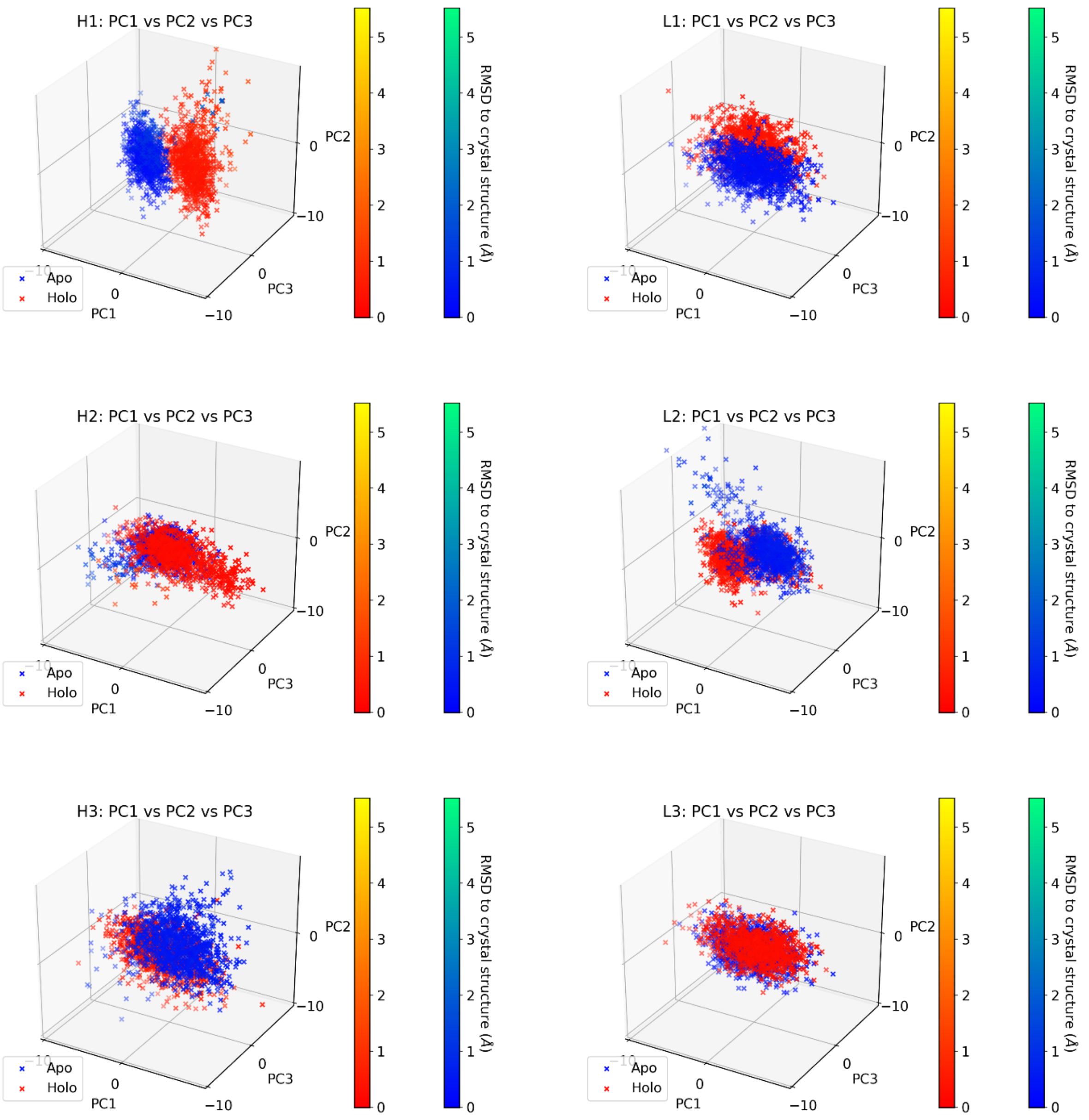
H1: PC1 vs PC2 vs PC3
L1: PC1 vs PC2 vs PC3
H2: PC1 vs PC2 vs PC3
L2: PC1 vs PC2 vs PC3
H3: PC1 vs PC2 vs PC3
L3: PC1 vs PC2 vs PC3
Apo
Holo
PC1
PC2
PC3
−10
0
RMSD to crystal structure (Å)
0
1
2
3
4
5

Anti-IL-18

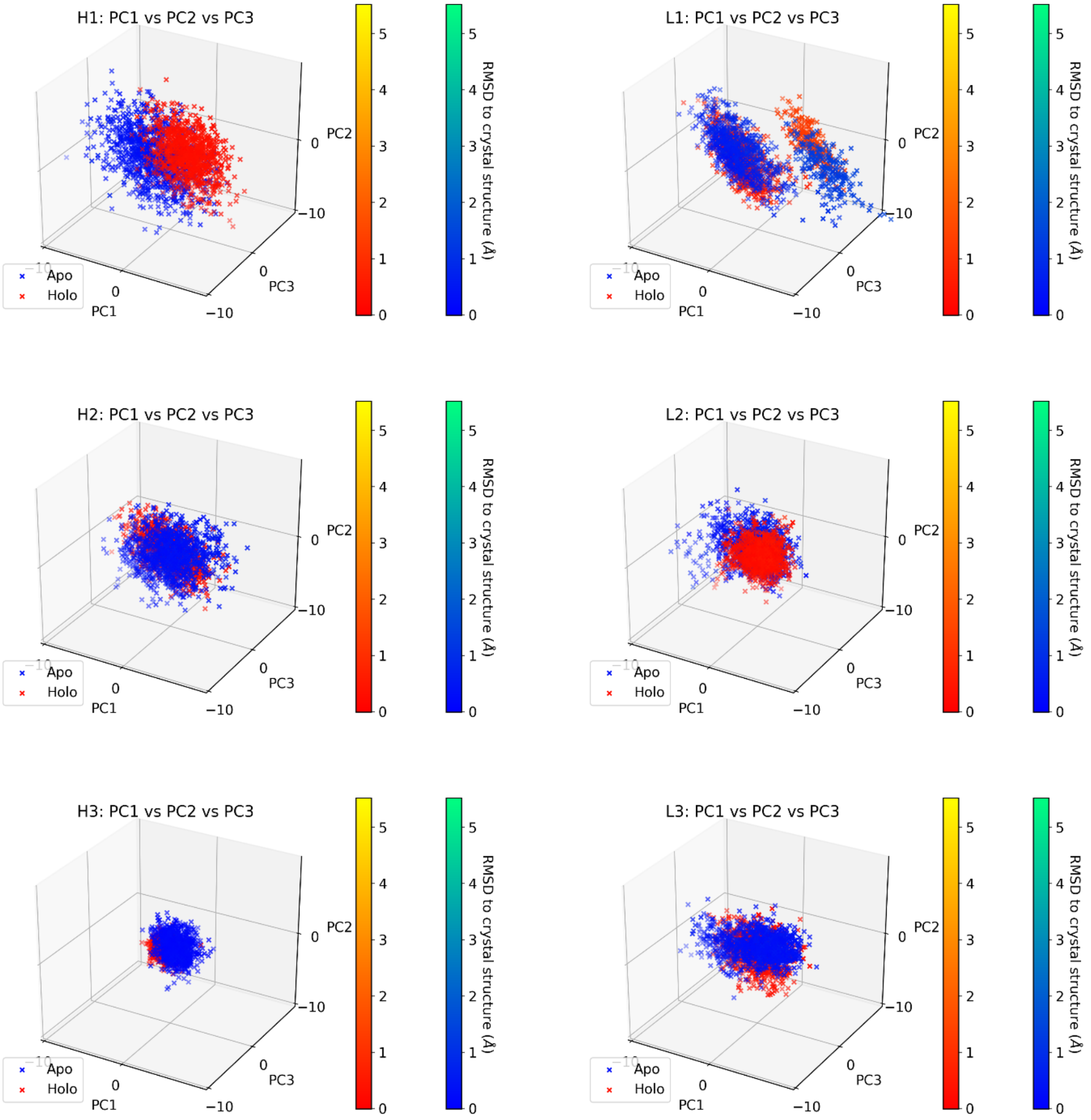
H1: PC1 vs PC2 vs PC3
L1: PC1 vs PC2 vs PC3
H2: PC1 vs PC2 vs PC3
L2: PC1 vs PC2 vs PC3
H3: PC1 vs PC2 vs PC3
L3: PC1 vs PC2 vs PC3
Apo
Holo
PC1
PC2
PC3
RMSD to crystal structure (Å)

Anti-LFA

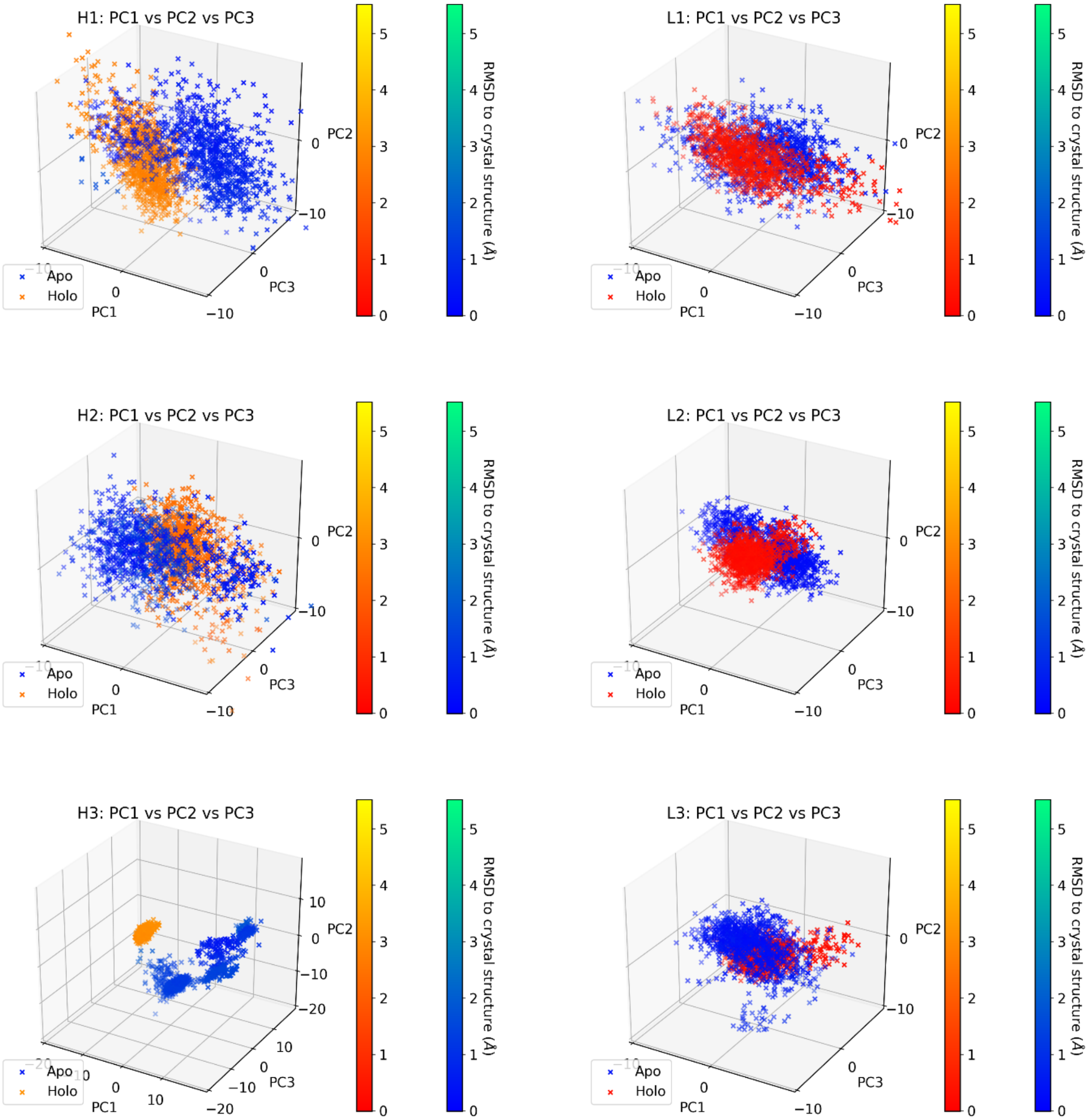
H1: PC1 vs PC2 vs PC3
L1: PC1 vs PC2 vs PC3
H2: PC1 vs PC2 vs PC3
L2: PC1 vs PC2 vs PC3
H3: PC1 vs PC2 vs PC3
L3: PC1 vs PC2 vs PC3
Apo
Holo
PC1
PC2
PC3
RMSD to crystal structure (Å)

Anti-MHC

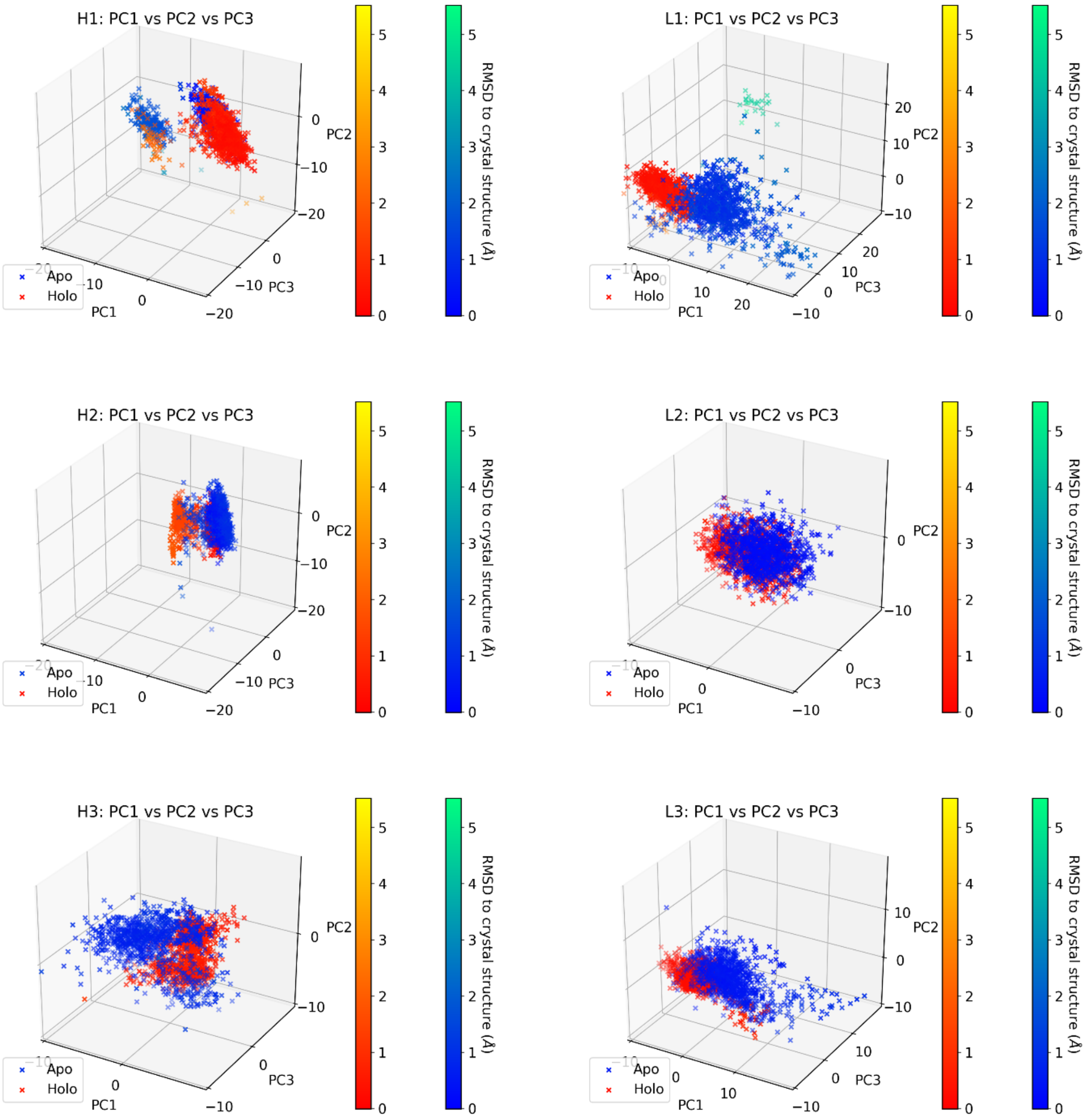
H1: PC1 vs PC2 vs PC3
L1: PC1 vs PC2 vs PC3
H2: PC1 vs PC2 vs PC3
L2: PC1 vs PC2 vs PC3
H3: PC1 vs PC2 vs PC3
L3: PC1 vs PC2 vs PC3
Apo
Holo
PC1
PC2
PC3
RMSD to crystal structure (Å)

Anti-ObR

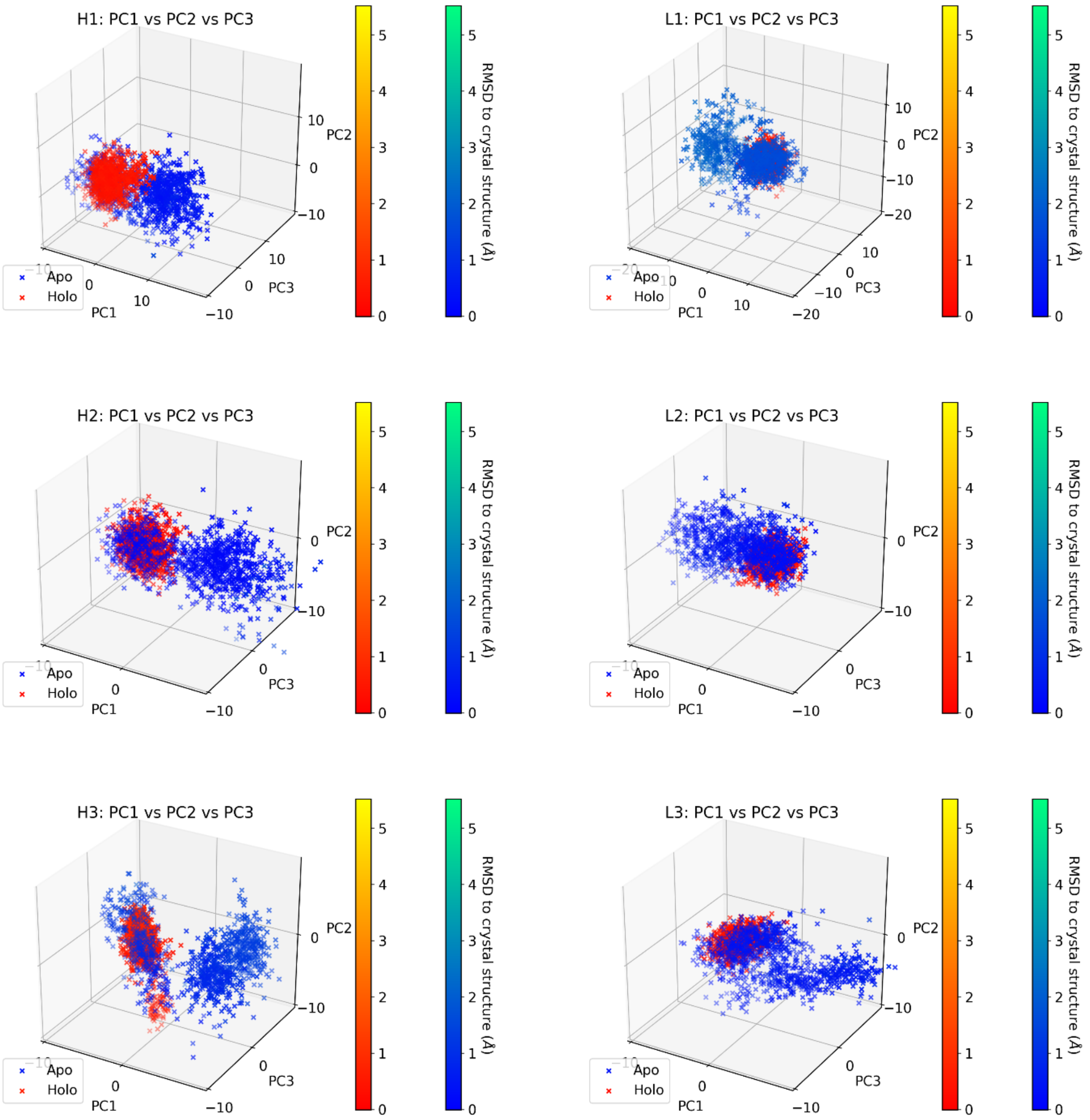
H1: PC1 vs PC2 vs PC3
L1: PC1 vs PC2 vs PC3
H2: PC1 vs PC2 vs PC3
L2: PC1 vs PC2 vs PC3
H3: PC1 vs PC2 vs PC3
L3: PC1 vs PC2 vs PC3
PC1
PC2
PC3
Apo
Holo
RMSD to crystal structure (Å)

Anti-IL-1βa

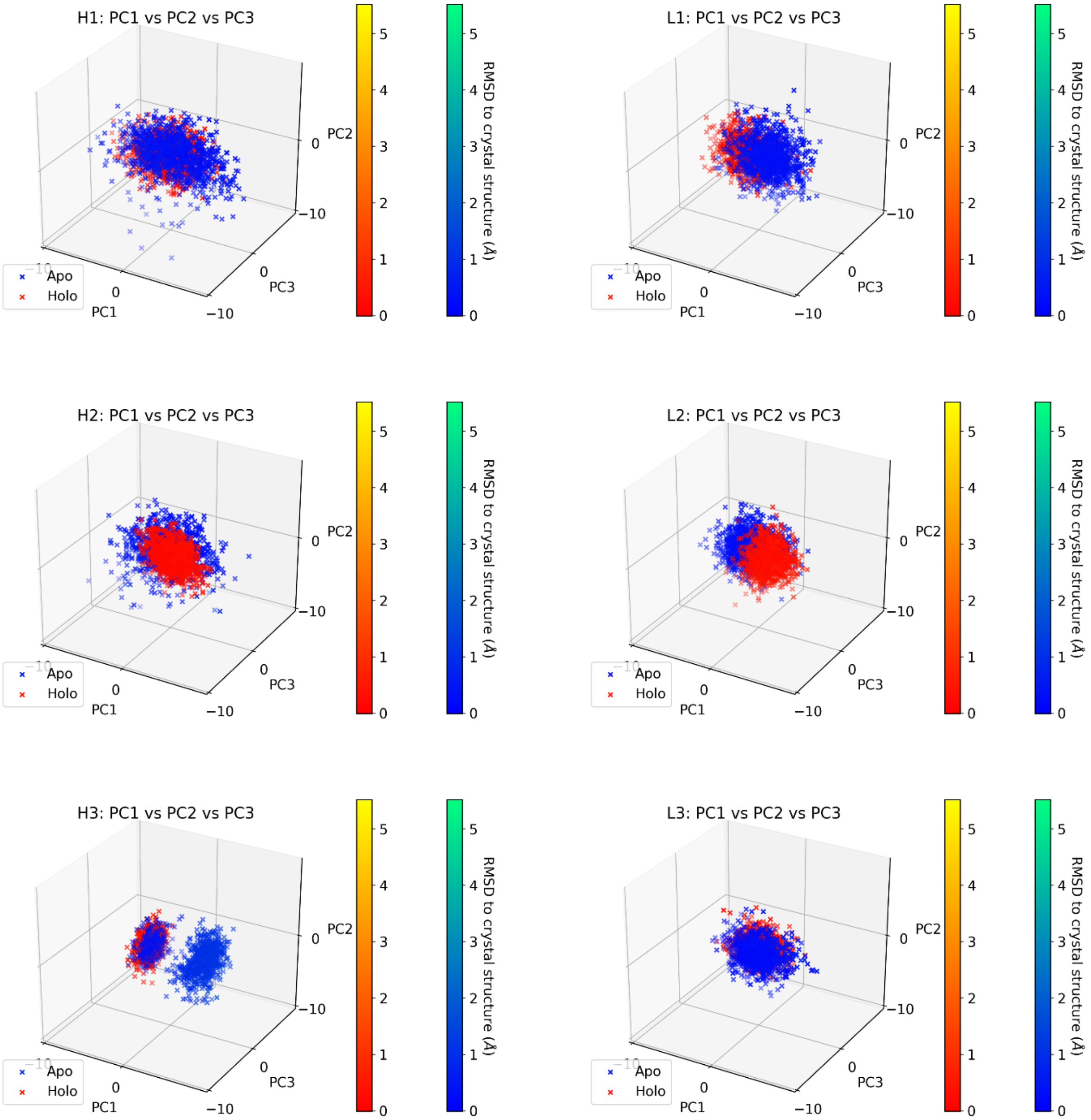
H1: PC1 vs PC2 vs PC3
L1: PC1 vs PC2 vs PC3
H2: PC1 vs PC2 vs PC3
L2: PC1 vs PC2 vs PC3
H3: PC1 vs PC2 vs PC3
L3: PC1 vs PC2 vs PC3
Apo
Holo
PC1
PC2
PC3
RMSD to crystal structure (Å)

Anti-TNFα

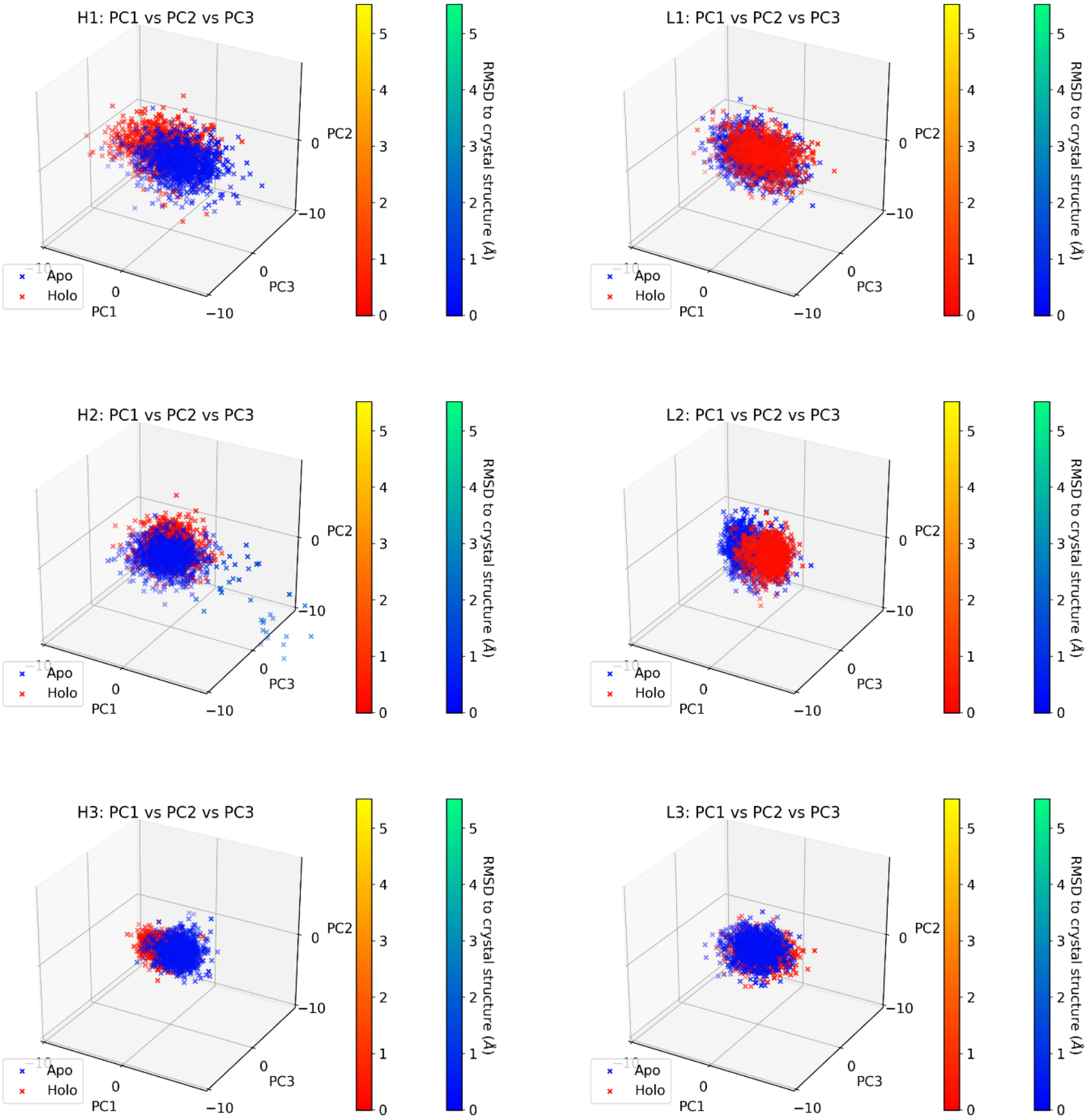
H1: PC1 vs PC2 vs PC3
L1: PC1 vs PC2 vs PC3
H2: PC1 vs PC2 vs PC3
L2: PC1 vs PC2 vs PC3
H3: PC1 vs PC2 vs PC3
L3: PC1 vs PC2 vs PC3
PC1
PC2
PC3
Apo
Holo
RMSD to crystal structure (Å)

Anti-IL-1βb

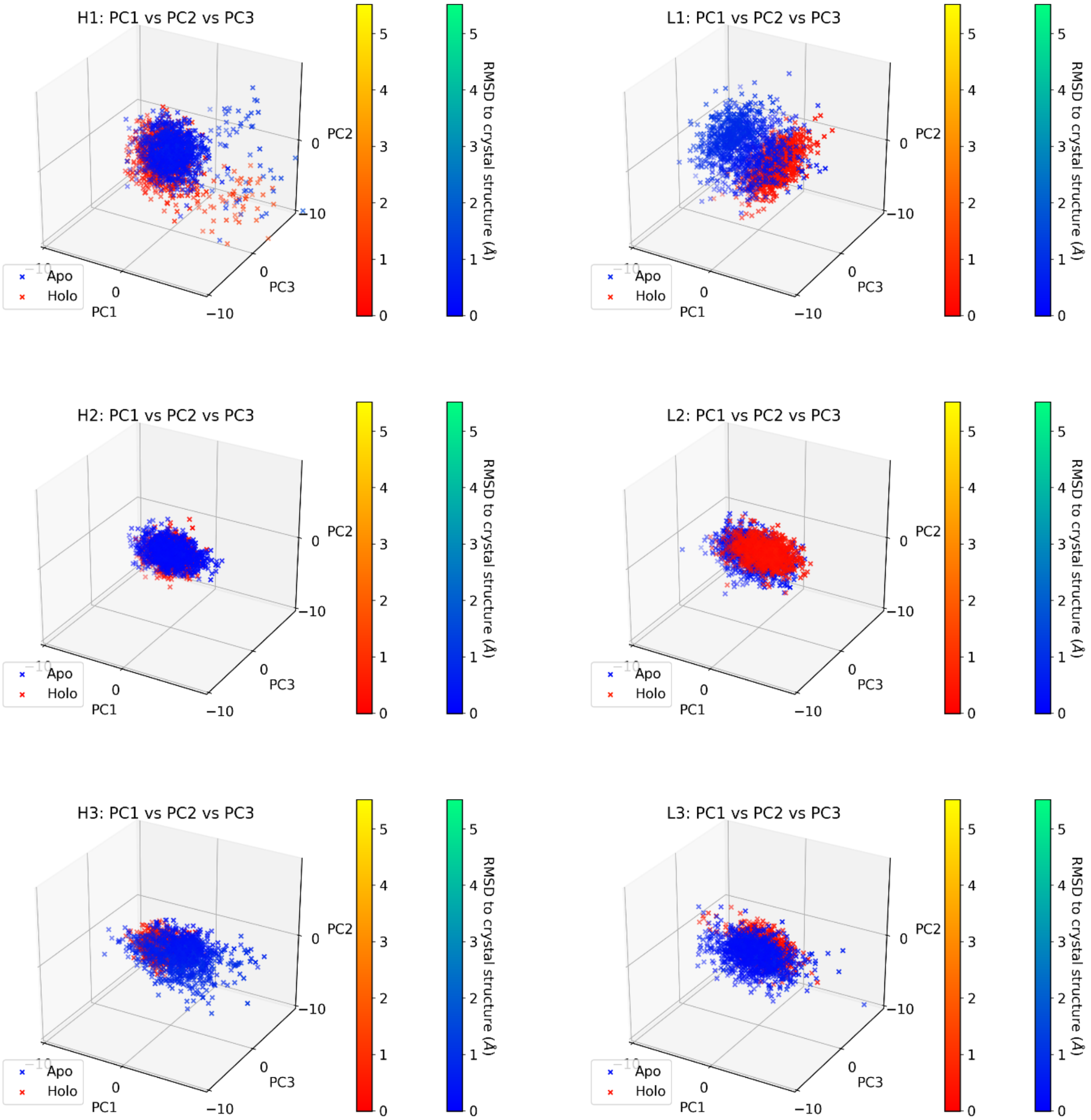
H1: PC1 vs PC2 vs PC3
L1: PC1 vs PC2 vs PC3
H2: PC1 vs PC2 vs PC3
L2: PC1 vs PC2 vs PC3
H3: PC1 vs PC2 vs PC3
L3: PC1 vs PC2 vs PC3
Apo
Holo
PC1
PC2
PC3
RMSD to crystal structure (Å)

Anti-PD

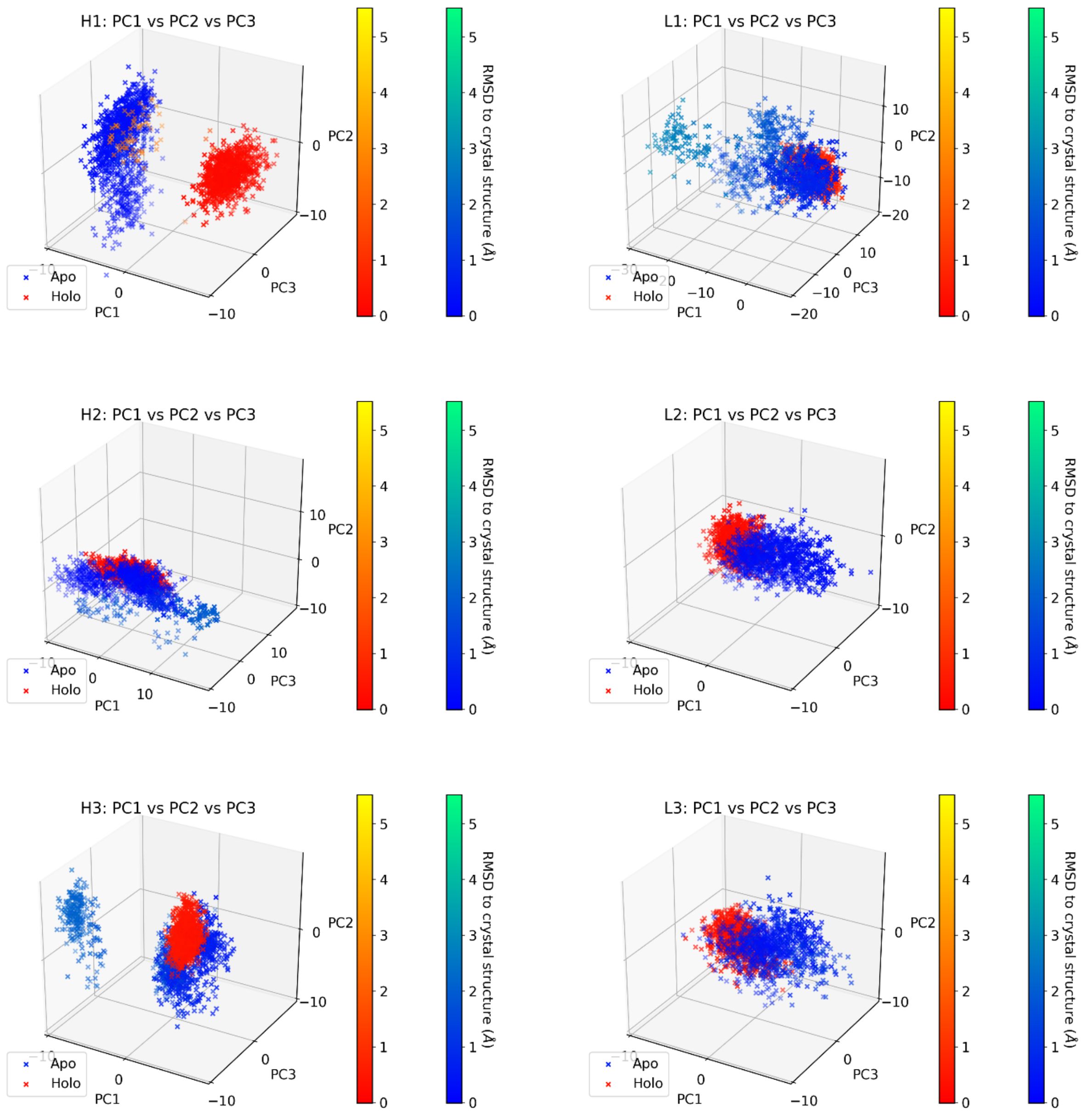

Supplementary Figure 2. Frames projected along PCs 1-3 for each CDR. Frames are colored by RMSD with respect to their crystal structure, with apo frames plotted in blue/green and holo frames plotted in red/yellow.

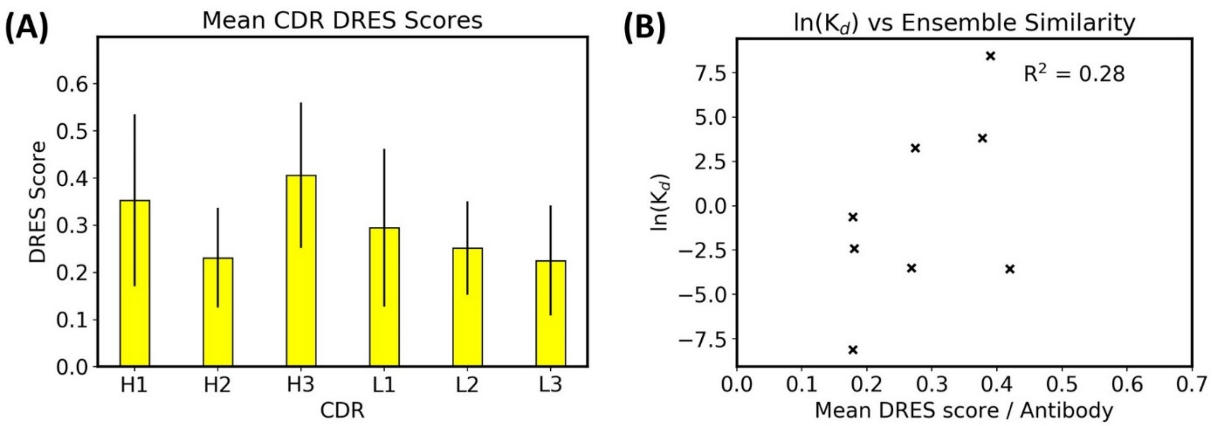

Supplementary Figure 3. DRES results. (A) Mean DRES scores for each CDR across the antibody-antigen dataset. Error bars are given to one standard deviation. (B) Mean DRES score for each antibody plotted against its experimental affinity, showing no correlation.

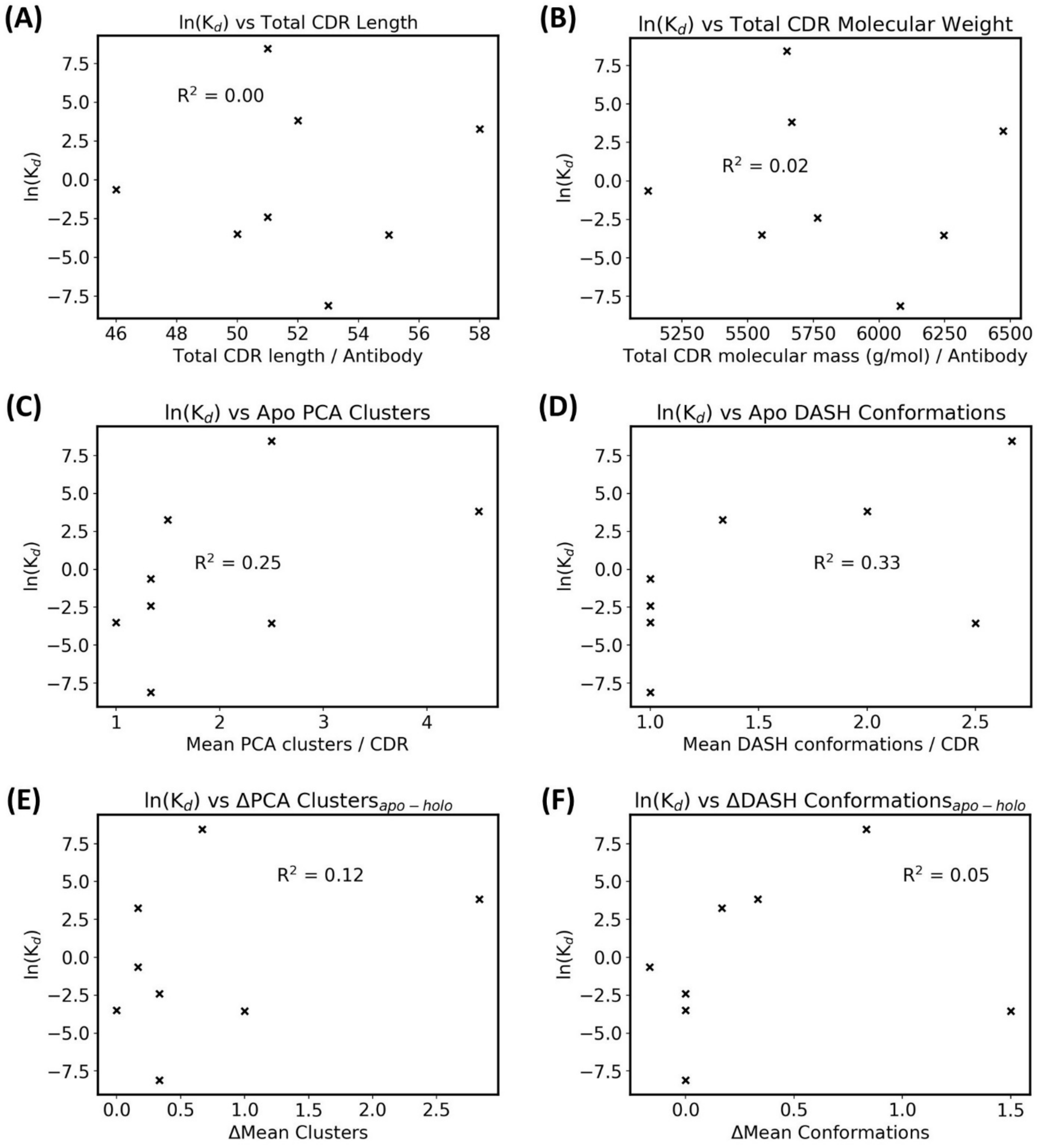

Supplementary Figure 4. Affinity against CDR characteristics. $\ln(K_d)$ is used as a proxy for affinity due to the Gibbs free energy equation of $\Delta G^o = -RT\ln(K_{eq})$, and natural logarithms of K_d values in nM are used (see main manuscript Table 1). Each antibody's experimental affinity is plotted against (A) total CDR length, (B) total molecular weight, (C) mean apo PCA clusters, (D) mean apo DASH conformations, (E) difference between apo and holo PCA clusters, and (F) difference between apo and holo DASH conformations.

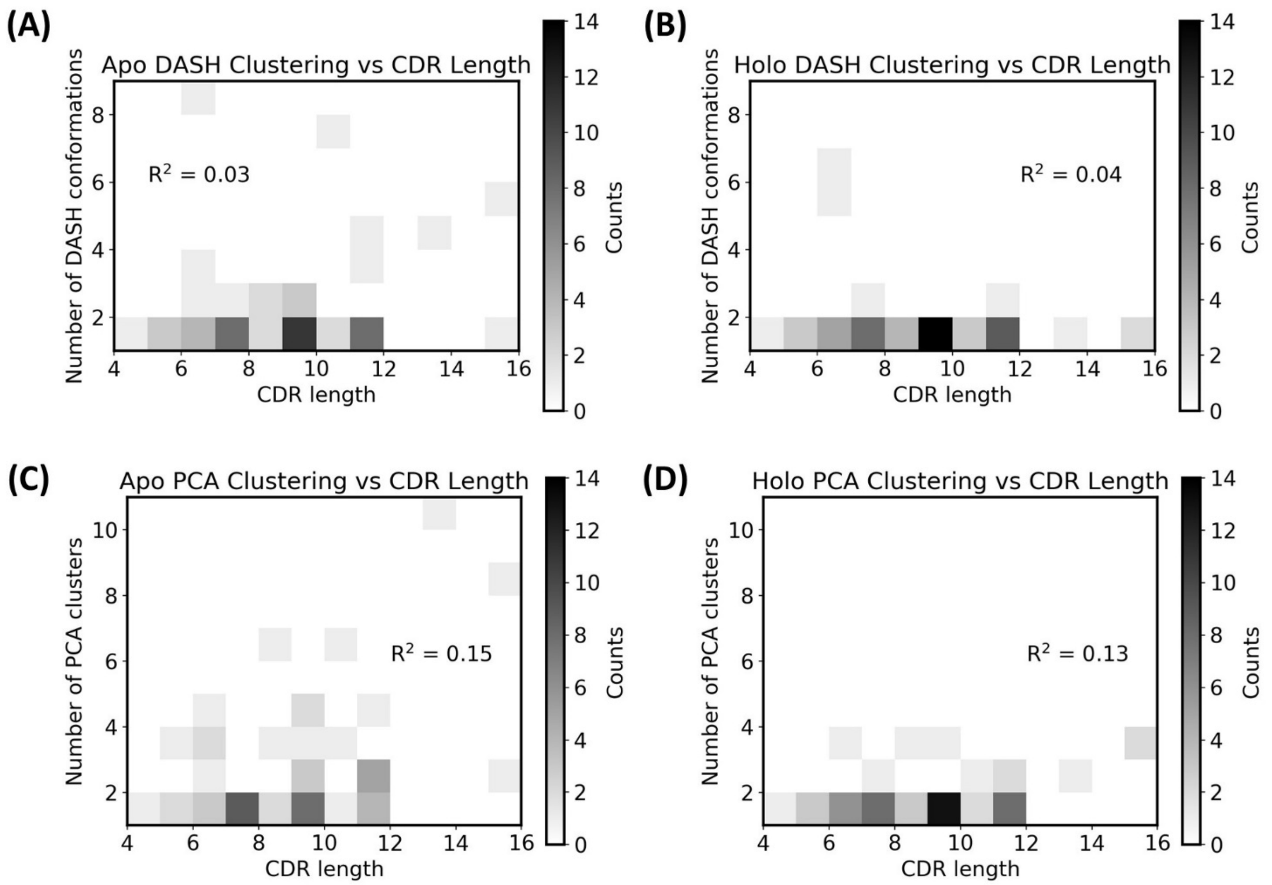

Supplementary Figure 5. Individual CDR clustering against CDR length. (A) and (B) plot CDR length against their DASH clusters, whereas (C) and (D) plot CDR length against PCA clusters.

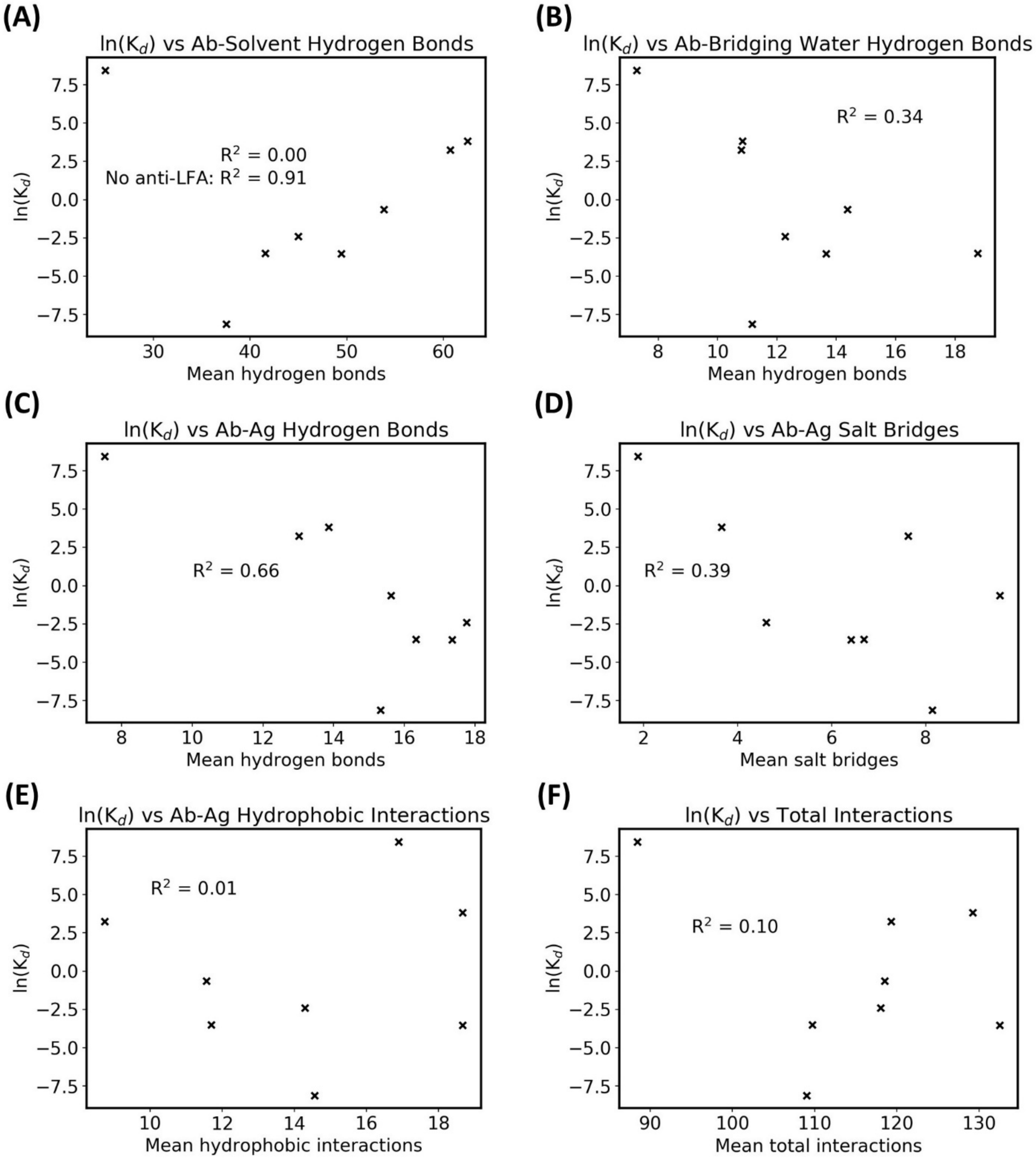

Supplementary Figure 6. Affinity vs antibody-antigen interaction counts for simulations using the fluctuating simulation interface.

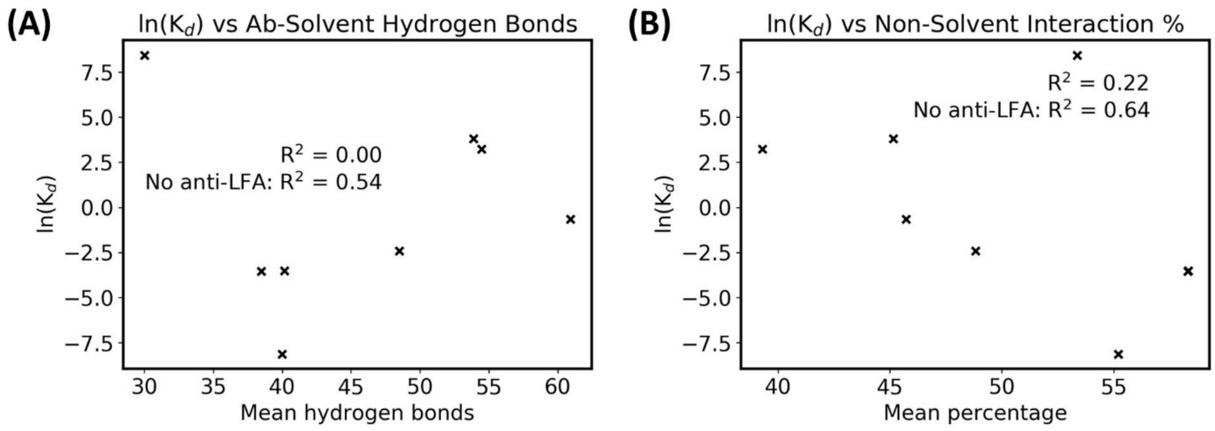

Supplementary Figure 7. Affinity vs antibody-solvent interactions for simulations using the crystal structure interface. (A) $\ln(K_d)$ against the mean number of hydrogen bonds that antibody interface atoms form with bulk solvent. (B) $\ln(K_d)$ against the percentage of antibody interface interactions that are not with bulk solvent. Note that there are two overlapping points at 58%.

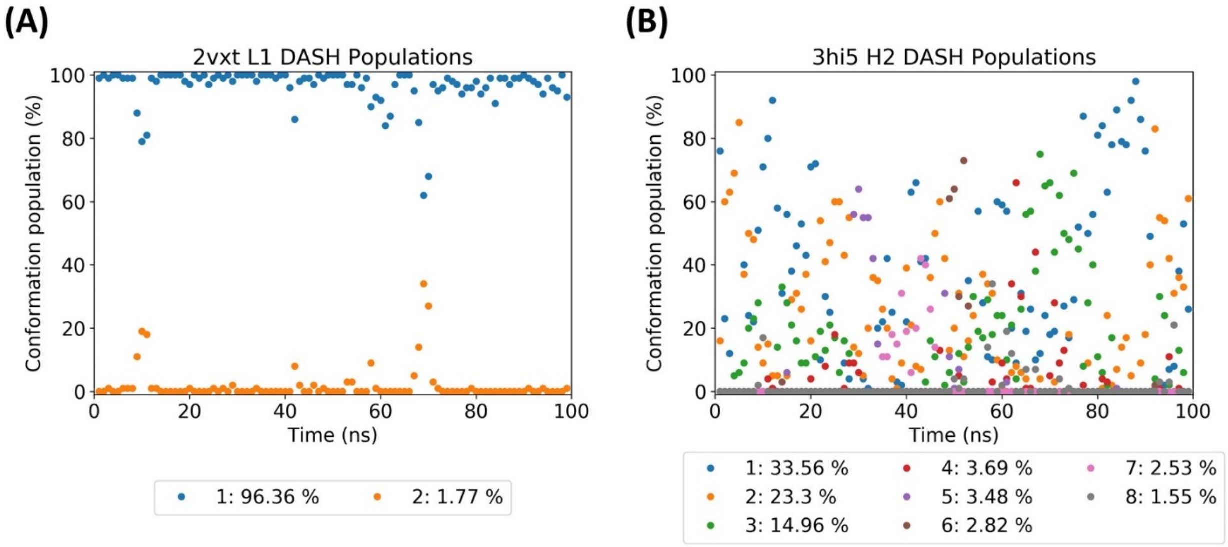

Supplementary Figure 8. Representative DASH population plots to check for convergence. Population is defined as the percentage of frames each DASH conformation occupies over a specified time period. Here the simulation trajectory is divided into a hundred 1 ns chunks, and the population for each conformation is calculated and plotted. (A) Example of a more converged CDR, where populations are relatively constant. (B) Example of a less converged CDR, where the populations show large variation.

2 Supplementary Tables

Supplementary Table 1. PCA hierarchical clustering counts.

Antibody	H1		H2		H3		L1		L2		L3	
	Apo	Holo	Apo	Holo	Apo	Holo	Apo	Holo	Apo	Holo	Apo	Holo
Anti-H1N1	2	1	1	1	2	3	2	1	1	1	1	1
Anti-IL-18	2	1	1	1	1	1	2	2	1	1	1	1
Anti-LFA	4	3	3	3	4	1	2	2	1	1	1	1
Anti-MHC	6	3	4	1	3	2	10	2	1	1	3	1
Anti-ObR	4	1	3	1	3	1	6	1	1	1	1	1
Anti-IL-1βa	1	1	1	1	1	1	1	1	1	1	1	1
Anti-TNFα	1	1	3	1	1	1	1	1	1	1	1	1
Anti-IL-1βb	1	1	1	1	1	1	2	1	1	1	2	1
Anti-PD	1	2	2	1	2	1	8	3	1	1	1	1

Supplementary Table 2. DASH dihedral clustering counts.

Antibody	H1		H2		H3		L1		L2		L3	
	Apo	Holo	Apo	Holo	Apo	Holo	Apo	Holo	Apo	Holo	Apo	Holo
Anti-H1N1	2	1	1	1	1	1	1	1	2	2	1	1
Anti-IL-18	1	1	1	1	1	1	1	2	1	1	1	1
Anti-LFA	1	1	8	6	3	1	1	1	1	1	2	1
Anti-MHC	2	1	3	5	1	1	4	1	1	1	1	1
Anti-ObR	1	1	1	1	2	1	7	1	1	1	1	1
Anti-IL-1βa	1	1	1	1	1	1	1	1	1	1	1	1
Anti-TNFα	1	1	1	1	1	1	1	1	1	1	1	1
Anti-IL-1βb	1	1	1	1	1	1	1	1	1	1	1	1
Anti-PD	1	1	2	1	4	1	5	1	1	1	2	1

Supplementary Table 3. DASH rare conformation percentages. The percentage of frames with rare states are summed for each CDR in this table.

Antibody	H1		H2		H3		L1		L2		L3	
	Apo	Holo	Apo	Holo	Apo	Holo	Apo	Holo	Apo	Holo	Apo	Holo
Anti-H1N1	0.12	3.46	0	0	0	0	0	0	0	0	0	0
Anti-IL-18	0	0	0	0	0	0	2.33	1.87	0	0	0	0
Anti-LFA	0	0	14.11	1.76	4.98	0	2.66	0	0	0	0.41	0
Anti-MHC	1.36	0	1.54	3.22	0	0	17.51	0	0	0	0	0
Anti-ObR	0	0	0	0	7.92	0	12.50	0	0	0	0	0
Anti-IL-1βa	0	0	0	0	0	0	0	0	0	0	0	0
Anti-TNFα	0	0	0	0	0	0	0	0	0	0	0	0
Anti-IL-1βb	0	0	0	0	0	0	0.04	0	0	0	0	0
Anti-PD	0	0	0	0	6.42	0	14.01	0	0	0	2.19	0.69

Supplementary Table 4. Apo and holo trajectories' matched conformation percentages. States from apo and holo were considered matched if their circular similarity score was at least 0.80, i.e. 80% similar or above.

Antibody	H1		H2		H3		L1		L2		L3	
	Apo	Holo	Apo	Holo	Apo	Holo	Apo	Holo	Apo	Holo	Apo	Holo
Anti-H1N1	0	0	100	100	100	100	100	100	100	100	100	100
Anti-IL-18	100	100	100	100	100	100	97.67	96.36	100	100	100	100
Anti-LFA	100	100	73.37	89.54	0	0	97.34	100	100	100	89.20	100
Anti-MHC	78.34	100	91.17	66.74	100	100	24.13	100	100	100	100	100
Anti-ObR	100	100	100	100	56.61	100	5.67	100	100	100	100	100
Anti-IL-1βa	100	100	100	100	100	100	100	100	100	100	100	100
Anti-TNFα	100	100	100	100	100	100	100	100	100	100	100	100
Anti-IL-1βb	100	100	100	100	100	100	99.96	100	100	100	100	100
Anti-PD	0	0	80.57	100	22.19	100	40.06	100	100	100	88.38	99.31

3 Supplementary Methods – starting structure selection

The REST2 dataset was selected in June 2018. Given there are fewer therapeutic antibody structures than non-therapeutic ones, the former was curated first:

- Starting with the seventy-two therapeutic antibodies listed on the SAbDab database, only twenty-six were solved in their apo and holo forms;
- Of these twenty-six antibody-antigen structures, only six had both structures of < 2.5 Å resolution;
- Of the six antibodies solved in their apo and holo forms at high resolution, one had missing residues in its CDRs and two had peptide antigens; these were discarded.

This left three structures for the dataset, and the non-redundant search results were considered next:

- SAbDab filters were set to ensure only antibodies and antigens with less than 70% sequence identity would be considered non-redundant, and that the antibody-antigen structure had a resolution of < 2.5 Å. This gave a total of forty-one antibodies;
- Of these forty-one holo antibody-antigen structures, only ten had the antibody solved in its apo form at < 2.5 Å resolution;
- Of the ten antibodies solved in its apo and holo form, only seven had no missing residues in either the CDRs or large portions of the antigen;
- Of the seven antibodies with no missing residues in important regions of interest, one was a therapeutic and already chosen for the dataset, one was anti-ObR which was the antibody used to optimise our protocol, and all the remaining antibodies had experimental affinities in the nM region.

Four non-therapeutic antibodies were chosen from these final six due to their similar experimental affinity. The seven chosen therapeutic and non-therapeutic antibodies had binding affinities ranging from fM to nM, and another antibody was purposely selected for its high µM affinity (AL-57, PDBs 3hi6 and 3hi5) to increase the range further.
